# First-principles density functional theoretical study on the structures, reactivity and spectroscopic properties of (NH) and (OH) Tautomer's of 4-(methylsulfanyl)-3[(1Z)-1-(2-phenylhydrazinylidene) ethyl] quinoline-2(1H)-one

**DOI:** 10.1038/s41598-023-35933-8

**Published:** 2023-06-01

**Authors:** Shimaa Abdel Halim, Mohamed A. Abdel-Rahman

**Affiliations:** 1 Chemistry Department, Faculty of Education, Ain Shams University, Roxy, Cairo, 11711 Egypt; 2 Chemistry Department, Faculty of Science, Suez University, Suez, 43518 Egypt

**Keywords:** Chemistry, Physical chemistry, Theoretical chemistry

## Abstract

The tautomerizations mechanism of 4-(methylsulfanyl)-3[(1Z)-1-(2-phenylhydrazinylidene) ethyl] quinoline-2(1H)-one were inspected in the gas phase and ethanol using density function theory (DFT) M06-2X and B3LYP methods. Thermo-kinetic features of different conversion processes were estimated in temperature range 273–333 K using the Transition state theory (TST) accompanied with one dimensional Eckert tunneling correction (1D-Eck). Acidity and basicity were computed as well, and the computational results were compared against the experimental ones. Additionally, NMR, global descriptors, Fukui functions, NBO charges, and electrostatic potential (ESP) were discussed. From thermodynamics analysis, the keto form of 4-(methylsulfanyl)-3-[(1Z)-1-(2 phenylhydrazinylidene) quinoline-2(1H)-one is the most stable form in the gas phase and ethanol and the barrier heights required for tautomerization process were found to be high in the gas phase and ethanol ~ 38.80 and 37.35 kcal/mol, respectively. DFT methods were used for UV–Vis electronic spectra simulation and the time-dependent density functional theory solvation model (TDDFT-SMD) in acetonitrile compounds.

## Introduction

Quinolinones, a quinoline heterocyclic analogue, have gotten a lot of attention for their physical, chemical, and biological activity as a treatment for sexually transmitted diseases, genitourinary infections, respiratory diseases, skin and soft tissue infections, prostate, and gastroenteritis^1^. Quinolinones have molluscicidal, fungicidal, and bactericidal activity; anti-HSV; anti-convulsion; anti-tumor; anti-oxidation; and anti-inflammatory activity^2–7^.

The position of the heterocyclic category depicted our consideration of the treatment of 3-acetyl-4-(methylsulfanyl)-quinolin-2(1H)-one with phenylhydrazine in boiling ethanol, which afforded a pale brown product that was identified as phenylhydrazone with a Z/E isomer ratio of 65:35. According to the literature^8^ elemental analysis revealed that compound 4-(methylsulfanyl)-3[(1Z)-1-(2-phenylhydrazinylidene) ethyl] quinoline-2(1H)-one lacked sulphur all at once.

Tautomeric phenomena occur when a molecule has multiple isomers. In organic chemistry, molecular biology, medicinal chemistry, and pharmacology, intramolecular H-atom transfer between two mixed equilibrium structures is extremely important. Tautomerism occurs in this work through the migration of an H-atom from one location to another. H-atom transfer produces keto-enol tautomers in the system under discussion. Tautomers are created when hydrogen atoms are exchanged between the N and O atoms of the heterocyclic ring^9,10^. Proton transport and H-bonding are important properties of the H-atom in chemistry. Theoretical research using the DFT approach, computational studies, and electrical, optical, and photoelectrical characteristics is very important to finding new drug candidates and understanding the electrical properties of different molecular structures^11–14^.

**Scheme 1:**
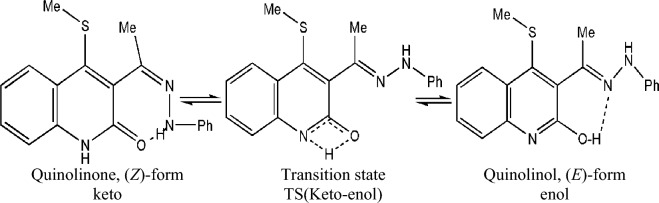
The tautomeric structure keto and enol-form of 4-(methylsulfanyl)-3[(1Z)-1-(2 phenylhydrazinylidene) ethyl] quinoline-2(1H)-one.

Due to lack of data of 4-(methylsulfanyl)-3[(1Z)-1-(2-phenylhydrazinylidene) ethyl] quinoline-2(1H)-one and the compound is partially soluble in water and completely soluble in ethanol^8^. We present here a computational study on the structures, relative stabilities, and electronic absorption spectra of tautomeric forms of 4-(methylsulfanyl)-3[(1Z)-1-(2-phenylhydrazinylidene) ethyl] quinoline-2(1H)-one (Scheme 1) in the gas phase and ethanol at B3LYP with 6-31G(d,p), 6-311++G(2d,2p) basis sets, and M06-2X/6-311++G(2d,2p) level. Proton transport and hydrogen bonding are two crucial factors that will be discussed.

There is a strong link between structure and stability. Understanding the chemical and physical features of these tautomers could aid future experimental studies on their expected applications, especially their ability to form metal complexes for analytical and biological purposes^15,16^. By comparing the resulting spectra against the experimental data, it is possible to understand the nature of the observed spectra and all its features^17^.

## Computational method

Becke's three-parameter and Lee–Yang–Parr hybrid functional (B3LYP) density functional theory (DFT) was used in conjunction with the 6-31G(d,p) basis set^18–20^ to fully optimize tautomer's and rotamers for their interconversions (detailed structures are given in Supplementary Table S1 in the Supporting information (SI) file). To characterize the nature of each stationary point on the potential energy surface, vibrational mode calculations were performed at the same level of optimization (B3LYP/6-31G(d,p) level). For more accurate energies, the B3LYP/6-31G (d, p) optimized structures were refined at the B3LYP/6-311++G (d, p) and meta-hybrid generalized gradient approximation M06-2X/6-311++G (d, p) levels of theories^21,22^.

For the transformation of keto—enol and enol—rotomar processes, rate coefficients were estimated in temperature range 273–333 K and calculated for the unimolecular reactions (*k*_*uni*_, in s^−1^) using the transition state theory (TST)^23–25^ at the high pressure (HP) limits. TST was recently used in many advanced studies^26–32^ and can be obtained from Eq. (1).

1 $$ k^{TST} (T) = \sigma \,\chi (T)\frac{{k_{B} T}}{h}\left( {\frac{RT}{{P^{o} }}} \right)^{\Delta n} \exp \left( { - \frac{{\Delta G^{o\dag } (T)}}{{k_{B} T}}} \right) $$

where *σ,* *χ*(*T*), *k*_*B*_*,* *T,* *h,* *R,* *P°,* and *G*^*o*^(*T*) refer to the reaction path degeneracy, tunneling correction, Boltzmann constant, temperature in Kelvin, Planck constant, ideal gas constant, the standard pressure and standard Gibbs free energy of activation for reaction, respectively. For unimolecular reactions, *Δn* = *0*.

Tunneling coefficient *χ*(*T*) was estimated using the 1D-Eckart tunneling correction (*Eck*)^33^ that previously mentioned in many studies^29–32^. 1D-Eckart tunneling correction can be obtained numerically by integration of probability of transmission (*p(E)*) over a Boltzmann distribution of energy as given in Eq. (2):

2 $$ \kappa_{{{\text{Eckart}}}} (T) = \frac{{\exp \left( {{{\Delta H_{f}^{ \ne ,0K} } \mathord{\left/ {\vphantom {{\Delta H_{f}^{ \ne ,0K} } {k_{{\text{B}}} T}}} \right. \kern-0pt} {k_{{\text{B}}} T}}} \right)}}{{k_{{\text{B}}} T}}\int_{o}^{\infty } {p(E)\exp \left( {{{ - E} \mathord{\left/ {\vphantom {{ - E} {k_{{\text{B}}} T}}} \right. \kern-0pt} {k_{{\text{B}}} T}}} \right)} \,dE $$

where *ΔH*_*f*_^*≠,0K*^ and is the zero-point corrected activation enthalpy in the forward direction.

The orbital interactions, atomic charges, and their effects on the structure and stability of the examined structures were calculated using the natural bond orbital (NBO) approach^34^ using NBO program version 3.1^35^.

Optimization was also performed in ethanol at the B3LYP/6-31G (d, p) level using the solvation model based on density (SMD)^36^. The solvation effect in ethanol was computed using B3LYP/6-311++G (2d, 2p) and M06-2X/6-311++G (2d, 2p) levels. The Gaussian 09W program^37^ was used to do all calculations. The study of tautomeric equilibria can be supported by nuclear magnetic resonance (NMR) spectroscopy. The gauge independent atomic orbital (GIAO) method was used to obtain NMR shielding relative to the 13C and 1H isotropic chemical shielding of tetramethyl silane (TMS) at the B3LYP/6-31G(d,p) optimized gas phase geometry in chloroform^38–40^. To recognize the stability and reactivity of the investigated structures, the global chemical reactivity descriptors were established from the highest occupied molecular orbital (HOMO) and lowest unoccupied molecular orbital (LUMO) energies^41–44^ consequently, electron affinity (*EA*), ionization potential (*IP*), absolute hardness (*η*), softness (*S*), electronegativity (*X*), chemical potential ($\mu$), electrophilicity index ($\omega )$ have been estimated in the gas phase at the B3LYP/6-31G (d, p) level of theory. The global chemical reactivity descriptors have been obtained from the following relations, Eqs. (3–9):

3 $$ EA = - E_{LUMO} $$

4 $$ IP = - E_{HOMO} $$

5 $$ \eta = \left( {E_{LUMO} - E_{HOMO} } \right)/2 $$

6 $$ S = 1/2\eta $$

7 $$ \chi = - \left( {E_{LUMO} + E_{HOMO} } \right)/2 $$

8 $$ \mu = \left( {E_{LUMO} + E_{HOMO} } \right)/2 $$

9 $$ \omega = \mu^{2} /2\eta $$

The Fukui function is one of the main density functional descriptors that are used to model chemical reactivity and intramolecular site selectivity^45^. Fukui functions condensed (CFF) *f*^+^(*r*), *f*^−^(*r*), *f*^*0*^(*r*) of investigated structures are computed using individual atomic charges for natural population analysis (NPA).

Fukui functions can be extracted from Eqs. (10–12):

10 $$ f^{ + } (r) \, = \, q_{r} \left( {N + 1} \right) \, - q_{r} \left( N \right) \, $$

11 $$ f^{0} (r) \, = \, q_{r} \left( {N + 1} \right) \, - q_{r} \left( {N - 1} \right) \, $$

12 $$ f^{ - } (r) \, = \, q_{r} \left( N \right) \, - q_{r} \left( {N - 1} \right) $$

where *f*^+^*(r),* *f*^*0*^(*r*), and *f*^-^(*r*) represent nucleophilic, radical, and electrophilic attacks. The dual descriptor *∆f*(*r*) resembles the difference between nucleophilic (Eq. (10)) and electrophilic attacks (Eq. (12)). For different sites, if *∆f*(*r*) > 0, this site tends to undergo a nucleophilic attack. Chemical reactivity towards negative and positive charges could also be expected through mapping electrostatic potential (ESP).

For the investigated structures, the electronic absorption spectra (EAS) have been inspected using time-dependent density functional theory (TD-DFT) and the Perdew, Burke, and Ernzerhof (PBE) method (abbreviated as TD-PBE) in acetonitrile via the SMD approach at the optimized gas phase geometry of B3LYP/6-31G(d,p), level^46,47^. The accurate PBE0 method can be used to estimate electron excitations for different dye compounds^48,49^. Though, for the inspected molecule compared to experimental results, it gives an underestimation of *λ*_*max*_ by 59 nm, whereas TD-PBE functionally overestimates and yields an 11–13 nm difference depending on basis sets. In order to draw the ultraviolet–visible (UV–Vis) spectra, Gauss Sum program^50^ was used. For more accurate results, the natural transition orbitals (NTOs)^51^ was investigated for different electrons excitations instead of the canonical orbitals. The NTOs were sketched using Gaussview^52^.

## Results and discussion

### Structural analysis

**Figure 1:**
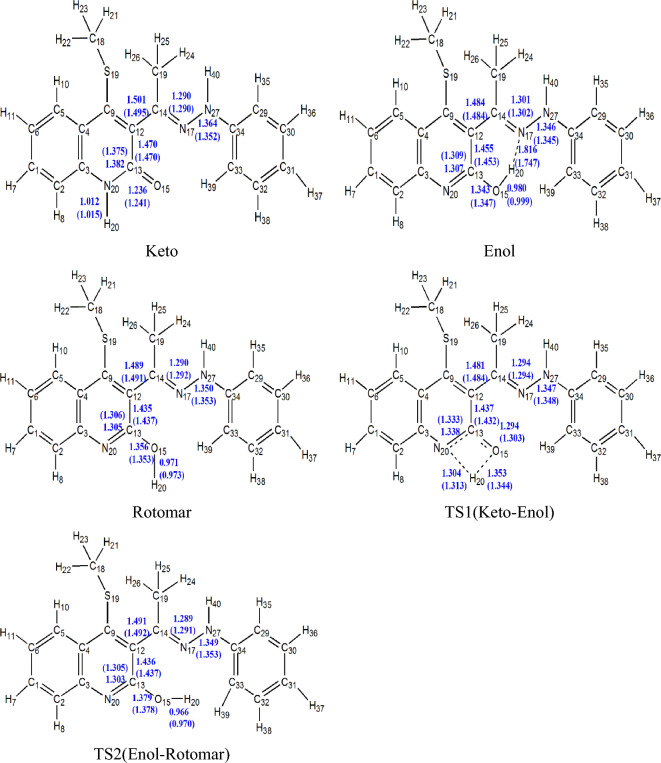
The optimized structures of keto-enol tautomers, transition state (TS) and rotamer (Rot) of 4-(methylsulfanyl)-3[(1Z)-1-(2 phenylhydrazinylidene) ethyl] quinoline-2(1H)-one in gas phase and ethanol (in parentheses) at B3LYP/6-31G(d,p) level of theory.

Previous studies demonstrated the accuracy of M06-2X functional in predicting the stability of tautomers and conformers^21,22,53,54^. Hence, the structures will be considered at B3LYP/6-31G (d, p) and energies at B3LYP/6-31G (d, p), B3LYP/6-311++G (d, p), and M06-2X/6-311++G (d, p). Three isomers of 4-(methylsulfanyl)-3[(1Z)-1-(2 phenylhydrazinylidene) ethyl] quinoline-2(1H)-one are discussed. Figure 1 shows the optimized structures of different structures at B3LYP/6-31G (d, p) level. To estimate and understand the stability order of the investigated system, reliable structures are necessary. A comparison between theory and experiment must be made to explain the reliability of the obtained data. The B3LYP/6-31G (d, p) level nearly reproduces the structure as obtained from X-ray^17^ and this supports the reliability of this level for structure optimization.

Scheme 1 depicts two tautomeric forms of 4-(methylsulfanyl)-3[(1Z)-1-(2 phenylhydrazinylidene) ethyl] quinoline-2(1H)-one with an intramolecular hydrogen bond (HB). The enol (Quinolinol, E-form) form indicates a phenol-imine HB (O–H···N, 1.816 Å) between the H- atom of the phenolic group and the N atom of the quinoline moiety. It is significant that the short donor–acceptor atom distance in both enol and keto forms provides a suggestion about the presence of a low-barrier hydrogen bond (LBHB), which is between 2.6 and 2.8 Å and has a noted biological importance^55–61^. The strength of HB in the tautomeric structures can be estimated from the donor (O_15_)-acceptor (N_17_) distance, which is 1.816 Å in enol form and 2.004 Å in keto form. Accordingly, the enol form has the strongest H-bond. In contrast, rotating the OH hydrogen atom to be far from the acceptor atom (nitrogen atom) gives the corresponding rotamer with no hydrogen bond and increases the donor–acceptor distance to 2.99 Å*.* Therefore, the keto-enol tautomers will be more stable than the rotamer.

### Thermodynamics and chemical kinetics

**Figure 2:**
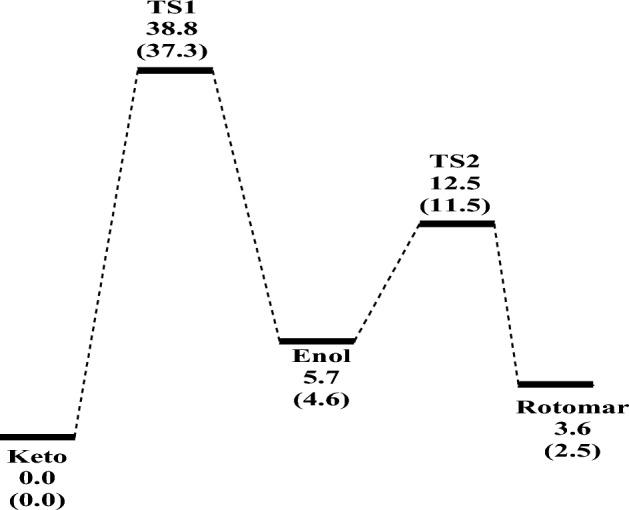
Potential energy diagram (*∆E*_*0K*_, *ΔE*_*0K*_^†^, in kcal/mol) for Keto → Enol (TS1) and Enol → Rotamer (TS2) conversions in gas phase and ethanol (in parentheses) using M06-2X energies.

Table 1 collects the barrier height and reaction energy for Keto–Enol reaction in gas phase and ethanol (in parentheses) at the B3LYP/6-31G (d, p) and M06-2X/6-311++G (2d, 2p)//B3LYP/6-31G (d, p) levels, while Fig. 2 shows the potential energy diagram using M06-2X energies. From M06-2X energies, the keto form is the most stable structure followed by rotamer in gas phase and in ethanol. The barrier heights required for the transformation process to the enolare 38.80 and 37.35 kcal/mol, respectively in gas phase and in ethanol, while the transformation of enol to rotamer accompanied barrier heights 12.54 and 11.54 kcal/mol and reaction energies 2.10 and 2.13 kcal/mol in gas phase and ethanol, respectively relative to keto form. The intrinsic reaction coordinates (IRC) and the potential energy changes during keto-enol conversion were drawn in Supplementary Figs. S1 and S2 (SI), respectively. From Supplementary Fig. S1, O–H bond is formed gradually with the breaking of the N–H bond and the two curves cross each other at s = 0 amu^1/2^ bohr. The formed N–C bond and the broken C–O bond are gently formed during the progress of the reaction.

**Table 1:** Thermodynamical parameters (in kcal/mol) of Keto → Enol (TS1) and Enol → Rotamer (TS2) conversions reactions in gas phase and ethanol (in parentheses) at the B3LYP/6-31G (d, p) and M06-2X/6-311++G (2d, 2p)//B3LYP/6-31G (d, p) levels.

Structure	B3LYP/6-31G (d, p)			M06-2X/6-311++G (2d, 2p)		
	*ΔE*_*0K*_^†^	*ΔH*_*298K*_^†^	*ΔG*_*298K*_^†^	*ΔE*_*0K*_^†^	*ΔH*_*298K*_^†^	*ΔG*_*298K*_^†^
Keto	0.00 (0.00)	0.00 (0.00)	0.00 (0.00)	0.00 (0.00)	0.00 (0.00)	0.00 (0.00)
TS1 (Keto–Enol)	35.47 (37.92)	35.61 (38.03)	34.85 (37.09)	38.80 (37.35)	38.94 (37.46)	38.18 (36.52)
Imaginary frequency TS1	1871.64i (1913.75i)					
Enol	6.07 (6.45)	6.13 (6.24)	5.67 (7.02)	5.73 (4.64)	5.79 (4.43)	5.33 (5.20)
TS2 (Enol–Rotomar)	14.75 (13.11)	14.91 (13.08)	13.95 (13.10)	12.54 (11.54)	12.72 (11.51)	11.76 (11.54)
Imaginary frequency TS2	430.97i (400.43i)					
Rotomar	4.80 (6.53)	5.09 (6.65)	3.99 (6.39)	3.63 (2.51)	3.93 (2.63)	2.82 (2.36)

The calculated rate coefficients for the selected transitions at *TST* and *1D-Eck* tunneling are given in Table 2. The results show that, the rate of the transformation of keto to enol in ethanol is higher about 12–20 times than in gas phase and a high effect for tunneling correction during the applied range of temperature especially for Keto–enol reaction compared to the enol-rotomar conversion.

**Table 2:** Lists *TST* rate coefficients of the conversion processes of Keto → Enol (TS1) and Enol → Rotamer (TS2) conversions over temperature range 273–333 K using *TST* approach and M06-2X energies.

*T* (*K*)	State	Keto → Enol (TS1)			Enol → Rotamer (TS2)		
		*TST*	*Eck*	*TST/Eck*	*TST*	*Eck*	*TST/Eck*
273	Gas phase	1.38 × 10^–18^	95,124.47	1.32 × 10^–13^	3.72 × 10^7^	1.26	4.70 × 10^7^
	Ethanol	2.85 × 10^–17^	148,662.31	4.24 × 10^–12^	4.31 × 10^7^	1.22	5.27 × 10^7^
283	Gas phase	1.82 × 10^–17^	26,294.35	4.77 × 10^–13^	6.06 × 10^7^	1.24	7.53 × 10^7^
	Ethanol	3.39 × 10^–16^	40,963.87	1.39 × 10^–11^	7.09 × 10^7^	1.21	8.55 × 10^7^
293	Gas phase	2.00 × 10^–16^	8519.58	1.70 × 10^–12^	9.57 × 10^7^	1.22	1.17 × 10^8^
	Ethanol	3.41 × 10^–15^	12,952.98	4.42 × 10^–11^	1.13 × 10^8^	1.19	1.34 × 10^8^
303	Gas phase	1.88 × 10^–15^	3111.18	5.84 × 10^–12^	1.46 × 10^8^	1.21	1.77 × 10^8^
	Ethanol	2.95 × 10^–14^	4662.48	1.37 × 10^–10^	1.74 × 10^8^	1.18	2.05 × 10^8^
313	Gas phase	1.53 × 10^–14^	1284.92	1.97 × 10^–11^	2.19 × 10^8^	1.19	2.61 × 10^8^
	Ethanol	2.22 × 10^–13^	1883.08	4.19 × 10^–10^	2.62 × 10^8^	1.17	3.06 × 10^8^
323	Gas phase	1.01 × 10^–13^	595.26	6.53 × 10^–11^	3.18 × 10^8^	1.18	3.77 × 10^8^
	Ethanol	1.48 × 10^–12^	848.14	1.25 × 10^–9^	3.85 × 10^8^	1.16	4.44 × 10^8^
333	Gas phase	6.99 × 10^–13^	302.65	2.11 × 10^–10^	4.54 × 10^8^	1.17	5.32 × 10^8^
	Ethanol	8.79 × 10^–12^	421.16	3.70 × 10^–9^	5.52 × 10^8^	1.15	6.33 × 10^8^

### NMR analysis

**Figure 3:**
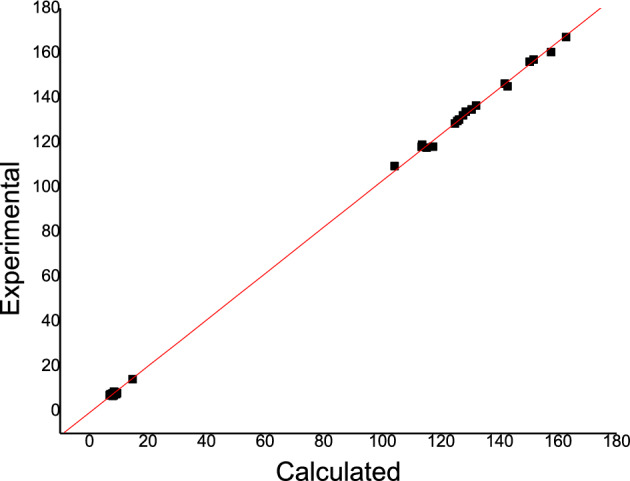
Sketches the experimental chemical shifts verses the estimated ^13^C and ^1^H NMR chemical shifts at the B3LYP/6-31G(d,p) level 4-(methylsulfanyl)-3[(1Z)-1-(2 phenylhydrazinylidene) ethyl] quinoline-2(1H)-one using the GIAO method in CHCl_3_.

In the NMR spectrum, the development of a low-field proton signal (high chemical shifts) is a well-known effect of forming a hydrogen bond with a sign for an LBHB^62–64^. Table 3 contains the complete data on NMR in enol, keto, and rotamer at B3LYP/6-31G(d,p) in comparison to experimental NMR. Figure 3 shows the calculated ^13^C and ^1^H NMR chemical shifts for 4-(methylsulfanyl)-3[(1Z)-1-(2 phenylhydrazinylidene) ethyl] quinoline-2(1H)-one in CHCl_3_ agree well with the experimental results^8^. Table 3 shows that C_18_ in the keto form has the highest chemical shift (171.33 ppm) that can be attributed to its closeness to the oxygen atom of the carbonyl group. Obviously, H_1_ of the keto form has the highest chemical shift (19.40 ppm) that attends the formation of strong LBHB (N_1_–H_1_…O_1_=C_2_), followed by H_1_ of the enol form (14.76 ppm). On the contrary, H_1_ of the rotamer has the lowest chemical shift (4.51 ppm). Hibbert and Emsley calculated ^1^H NMR chemical shifts to help predict the presence or absence of HB and distinguish LBHB for a proton chemical shift of up to 20 ppm (relative to TMS)^65^.

**Table 3:** ^13^C and ^1^H NMR chemical shifts (in ppm) calculated using the GIAO method in CHCl_3_ at the B3LYP/6-31G(d,p) level for 4-(methylsulfanyl)-3[(1Z)-1-(2 phenylhydrazinylidene) ethyl] quinoline-2(1H)-one.

^13^C chemical shifts	Enol	Exp	Keto	Rot
C1	162.75	167.00	156.25	164.82
C2	104.25	109.40	101.21	108.51
C3	130.51	134.70	128.62	129.31
C4	125.62	129.50	128.44	123.59
C5	132.04	136.50	129.69	131.38
C6	126.22	130.20	129.18	124.16
C9	128.53	133.70	118.29	130.98
C12	141.87	146.20	139.18	142.49
C13	150.37	156.00	143.42	153.35
C14	115.13	117.70	109.98	121.04
C18	157.64	160.30	171.33	150.67
C19	113.51	118.00	119.38	111.62
C29	127.59	132.10	129.82	125.40
C30	113.67	119.00	108.40	115.19
C31	124.81	128.40	124.55	128.62
C32	142.78	145.02	138.81	141.94
C33	151.73	157.00	144.24	152.53
C34	117.31	118.07	108.89	122.40
^1^H chemical shifts				
H7	7.83	7.70	7.16	7.68
H8	8.62	8.49	8.07	8.61
H10	7.88	7.63	7.64	7.80
H11	8.11	7.78	7.69	8.09
H21	7.96	7.73	7.67	7.91
H22	8.43	8.55	7.72	8.52
H23	7.03	7.10	6.65	6.74
H24	7.43	7.35	7.25	7.38
H25	7.01	6.96	6.43	7.12
H26	9.51	7.94	7.66	7.87
H35	8.30	7.01	6.56	6.47
H36	7.34	7.53	7.52	7.83
H37	8.01	6.69	6.34	7.21
H38	9.11	7.49	8.66	7.78
H39	8.38	7.07	8.61	7.86
H1-O/N	14.76	14.22	19.40	4.51
H40	7.03	7.10	6.65	6.74

### DFT reactivity descriptors and Fukui function

Table 2 shows the energies of LUMO, HOMO, the energy gap (*E*_*g*_), vertical *IP*, *EA*, $\eta$*,*$S$*,* $\chi$*,* $\mu$ and $\omega$. The *IP* represents the ability to donate an electron, while the *EA* illustrates the ability to accept an electron. According to Table 4, the keto form has the smallest HOMO–LUMO gap (3.00 eV), followed by rotamer (3.53 eV). The enol form has the highest gap (3.78 eV). The smaller the energy gaps, the higher the reactivity of the molecule^66,67^. So, the keto form is expected to have high chemical reactivity, low hardness, and high softness compared to enol and their rotamers. The keto form can act as an electron donor and acceptor by having the highest HOMO energy (*E*_*HOMO*_ = − 5.36 eV, the lowest value of the *IP*) and the lowest LUMO energy (*E*_*LUMO*_ = − 2.36 eV, the largest value of the affinity). However, based on the calculations, *ω* and $\eta$ they are better suited to act as strong electrophiles. A good electrophile has a high chemical potential as well as a low hardness^68^. Because the keto form has the highest electronegativity (*χ* = 3.86 eV) and the most negative chemical potential, it is the best electron acceptor.

**Table 4:** Global chemical descriptor (eV) of the studied structures at B3LYP/6-31G(d,p) level in the gas phase.

Structure	*E*_*HOMO*_	*E*_*LUMO*_	*E*_*g*_	*IP*	*EA*	*χ*	*η*	*S*	*μ*	*Ω*
Enol	− 5.53	− 1.75	3.78	5.53	1.75	3.64	1.89	0.26	− 3.64	3.51
Keto	− 5.36	− 2.36	3.00	5.36	2.36	3.86	1.50	0.33	− 3.86	4.97
Rotamer	− 5.21	− 1.68	3.53	5.21	1.68	3.45	1.77	0.28	− 3.45	3.37

To determine the most favorable place to add or remove an electron from a molecule, one looks at the chemical reactivity, which is one of the most fundamental questions. Electron density distributions are basic to understanding electrophilic and nucleophilic attacks. Table 5 shows the calculated condensed Fukui function value for enol and keto forms, and values for rotamers are given in the supporting information.

**Table 5:** Condensed Fukui functions (*f*_*k*_^+^*,* *f*_*k*_^*−*^*,* *f*_*k*_^*0*^) and dual-descriptor (*Δf*_*k*_) evaluated from Natural Population Analysis for Enol, Keto and Rotamer forms at B3LYP/6-31G(d,p).

Enol					Keto					Rotamer				
*Z*^*a*^	*f*_*k*_^*−*^	*f*_*k*_^+^	*f*_*k*_^*0*^	(*Δf*_*k*_)	*Z*^*a*^	*f*_*k*_^*−*^	*f*_*k*_^+^	*f*_*k*_^0^	(*∆f*_*k*_)	*Z*^*a*^	*f*_*k*_^*−*^	*f*_*k*_^+^	*f*_*k*_^*0*^	(*∆f*_*k*_)
N17	0.0374	− 0.0937	0.0189	0.0563	N29	0.0239	− 0.0802	0.0324	0.0563	N17	0.0329	− 0.070	0.1071	0.0371
C12	0.0222	0.0067	0.0144	− 0.0155	C12	0.0013	0.0276	0.0144	0.0264	C12	0.0293	0.0356	0.0293	0.0063
C13	0.0258	0.0159	0.0060	0.0099	C13	0.0188	0.0229	0.0147	0.0041	C13	0.0287	0.0329	0.0371	0.0042
C14	0.0327	0.0563	0.0445	0.0236	C14	0.0461	0.0428	0.0445	− 0.0033	C14	0.0482	0.0583	0.0381	− 0.0101
C5	0.0348	0.0163	0.0255	− 0.0185	C5	0.0207	0.0304	0.0255	0.0097	C5	0.0374	0.0340	0.0366	0.0034
C6	0.0406	0.0694	0.0550	0.0288	C6	0.0595	0.0506	0.0550	− 0.0089	C6	0.0695	0.0706	0.0510	− 0.0011
C9	0.0333	0.0123	0.0228	− 0.0210	C9	0.0118	0.0339	0.0228	0.0221	C9	0.0381	0.0229	0.0077	0.0152
C4	0.0077	0.0564	0.0320	0.0487	C4	0.0499	0.0142	0.0320	− 0.0357	C4	0.0549	0.0242	0.0310	− 0.0307
H7	0.0250	0.0233	0.0242	− 0.0017	H7	0.0172	0.0311	0.0242	0.0139	H7	0.0185	0.0411	0.0242	0.0226
H8	0.0239	0.0246	0.0242	0.0007	H8	0.0213	0.0272	0.0242	0.0059	H8	0.0273	0.0182	0.0276	0.0091
H10	0.0267	0.0277	0.0272	0.0010	H10	0.0247	0.0297	0.0272	0.0049	H10	0.0317	0.0267	0.0372	0.0050
H11	0.0268	0.0281	0.0274	0.0013	H11	0.0249	0.0300	0.0274	0.0051	H11	0.0369	0.0250	0.0380	0.0119
S16	0.0256	0.0266	0.0261	0.0011	S27	0.0232	0.0289	0.0261	0.0057	S16	0.0432	0.0389	0.0475	0.0043
C12	0.0203	0.0197	0.0200	− 0.0006	C12	0.0147	0.0254	0.0200	0.0107	C12	0.0247	0.0354	0.0200	0.0107
C13	0.0046	0.0120	0.0028	0.0074	C13	− 0.0128	0.0294	0.0128	0.0166	C13	− 0.0228	0.0194	0.0128	0.0166
C15	0.0093	0.0285	0.0477	0.0192	C28	0.0425	− 0.0047	0.0331	− 0.0378	C15	0.0625	− 0.045	0.0831	− 0.0175
C16	− 0.0174	− 0.0567	0.0960	0.0393	C29	− 0.0706	− 0.0035	0.0636	− 0.0671	C16	− 0.0206	− 0.035	0.0062	− 0.0144
C17	− 0.0099	0.0292	0.0485	0.0193	C30	0.0002	0.0190	0.0096	0.0188	C17	0.0084	0.0270	0.0096	0.0186
C18	0.0361	0.0367	0.0364	0.0005	C31	0.0350	0.0378	0.0364	0.0029	C18	0.0450	0.0578	0.0264	0.0128
C19	0.0300	0.0516	0.0408	0.0215	C32	0.0399	0.0417	0.0408	0.0017	C19	0.0599	0.0317	0.0308	0.0282
C20	0.0220	0.0156	0.0188	− 0.0063	C33	0.0264	0.0112	0.0188	− 0.0152	C20	0.0624	0.0442	0.0260	− 0.0182
C14	0.0250	0.0316	0.0283	0.0066	C14	0.0283	0.0283	0.0283	0.0000	C14	0.0303	0.0183	0.0283	0.0120
N17	0.0253	0.0295	0.0337	0.0042	N28	0.0281	0.0267	0.0281	− 0.0014	N17	0.0181	0.0367	0.0281	− 0.0186
N27	0.0156	0.0229	0.0193	0.0073	N29	0.0203	0.0183	0.0163	− 0.0020	N27	0.0503	0.0483	0.0163	− 0.0020
O15	− 0.0194	− 0.0576	0.0194	0.0382	O26	− 0.0319	− 0.0452	0.0319	0.0133	O15	− 0.0229	− 0.045	0.0319	0.0221
H28	0.0031	0.5219	0.2625	0.5188	H39	0.0388	0.4862	0.2625	0.4474	H28	0.0288	0.2412	0.2625	0.2124

^a^Atom numbering is given in Fig. 1.

From Table 5 values, the electrophilic attack order for enol in the gas phase is C6 > N17 > C4 > C5 > C9 > C14 > C13 > C12. The C6 and N17 atoms have a higher *f*^*–*^ value, indicating possible electron acceptor sites. There is some evidence that reactive electrophilic sites are primarily found on the hydrazinylidene ring. Conversely, for the nucleophilic attack, the reactivity order is C6 > C4 > C14 > C33 > C34. According to the dual descriptor (*∆f*) > *0* value for N17 and O15, these sites are favored for nucleophilic attack. As well as all hydrogen atoms, H is highly nucleophilic and H7 is highly electrophilic.

The C6, C4, C14, C12, N29, C5, C13, and C9 atoms in keto form are more sensitive sites for accepting electrons, and the C6, C14, C9, C5, C12, C13, C20, and C4 atoms are the most favorable sites for electron donation. Hence, heterocyclic rings are the most reactive sites for electron donor–acceptor interactions. The C20 and N29 atoms have a very positive dual descriptor (*∆f*) value, indicating a proclivity to donate electrons. According to the highly negativity dual descriptor (*∆f*), the C20 and C4 atoms are the most favorable sites for accepting electrons. Because it has the highest values of *f*^*−*^ and *f*^+^ in both enol and keto forms, the C6 atom is a suitable site for both electrophilic and nucleophilic attacks.

For rotamer form, it is observed that C9, C5, C12, C13, C14, and C6 atoms have a higher *f*^–^ value, which shows the possible site for electrophilic attack, and C6, C12, C4, C13, and C14 have a higher *f*^+^ value which indicates the possible site for nucleophilic attack. The dual descriptor (*∆f*) values of C12, C13, and C6 are highly positive; they have the tendency to donate electrons. On the contrary, N17 and O15 are susceptible to acquiring electrons.

### Charge distribution and ESP analysis

The electrostatic potential (ESP) and charge distribution surface are broadly used to determine the reactivity of a given molecule and its expected interaction with other systems. The NBO charges of the investigated structures have been calculated in Table 6 in the gas phase at the B3LYP/6-31G (d, p) level. In agreement with the Fukui function results, the NBO charges calculations for 4-(methylsulfanyl)-3[(1Z)-1-(2 phenylhydrazinylidene) ethyl] quinoline-2(1H)-one show that higher negative charges are located on the N and O atoms and a higher positive charge is obtained by H. Three carbon atoms (C12, C13, and C14) have a positive charge due to the presence of highly electronegative nitrogen and oxygen atoms. Table 6 showing the enol form has the highest negative charges on the O and N atoms with the highest electronegativity. Thus, the enol form has the highest potential to act as a bidentate ligand.

**Table 6:** NBO charges of all atoms^a^ of the investigated systems calculated at the B3LYP/6-31G(d,p) level of theory in the gas phase.

Atom	Enol	Keto	Enol–Rotamer
N	− 0.557	− 0.543	− 0.466
C12	0.229	0.250	0.213
C13	− 0.292	− 0.285	− 0.300
C14	− 0.066	− 0.061	− 0.075
C4	− 0.173	− 0.186	− 0.170
C5	− 0.239	− 0.225	− 0.248
C6	− 0.210	− 0.228	− 0.206
C9	− 0.233	− 0.212	− 0.245
S16	− 0.194	− 0.236	− 0.182
C15	0.212	0.229	0.188
C16	− 0.169	− 0.202	− 0.125
C17	0.386	0.439	0.368
C18	− 0.293	− 0.303	− 0.319
C19	− 0.207	− 0.206	− 0.214
C20	− 0.277	− 0.287	− 0.270
N27	− 0.188	− 0.192	− 0.195
O	− 0.701	− 0.688	− 0.667
H7	0.522	0.486	0.489
H8	0.244	0.252	0.241
H10	0.246	0.248	0.243
H11	0.246	0.248	0.243
H28	0.245	0.247	0.241
H39	0.248	0.250	0.244
H40	0.257	0.258	0.258
H23	0.250	0.245	0.231
H24	0.240	0.237	0.241
H25	0.240	0.237	0.241
H26	0.233	0.229	0.241

^a^Atom numbering is given in Fig. 1.

**Figure 4:**
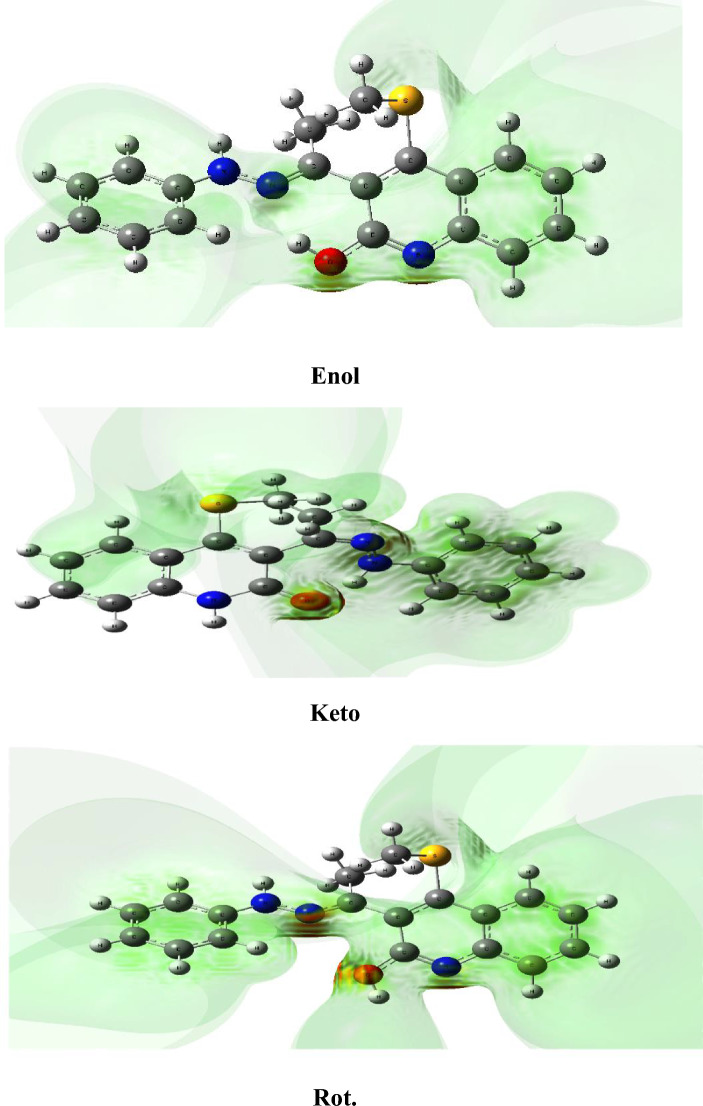
Molecular ESP surfaces of the investigated structures at B3LYP/6-31G(d,p).

ESP maps are described by the charged regions in the molecule. The different colours represent different values of the electrostatic potential. The potential grows in the following order: red, orange, yellow, green, and blue. The red colour in ESP maps represents the most negative electrostatic potential, while the blue colour reflects the most positive electrostatic potential regions. The ESP surfaces of investigated structures obtained using B3LYP/6-31G(d,p) are shown in Fig. 4. In the enol and keto forms, the ESP show the localization of a significant negative charge on the O atom, while the blue colour appears around the H7 and H8 atoms of the phenylhydrazinylidene ring. Therefore, the O atom has the highest electron donation ability toward metal ions. A significant blue colour on the H attached to O atoms in rotamers has been observed, and the red colour that exists in the region between the N and O atoms is due to the decline of H-bonds.

### Natural charge and natural population analysis

The natural population analysis (NPA) carried out on the electronic structures of the selected transition states to describe the distribution and arrangement of different electrons in various sub-shells atomic orbits. The accumulation of charges on the atom and the accumulation of electrons in the core, valence and rydberg sub-shells are summarized in Supplementary Figs. S3–S5 and Supplementary Tables S2–S3 in SI file for the keto–enol conversion in the gas phase and ethanol (in parentheses). According to Supplementary Tables S2 and S3, the most electronegative center charge are accumulated on O15, N17, N20 and N27 atoms − 0.72488 (− 0.72666), − 0.25119 (− 0.24709), − 0.62136 (− 0.61801), and − 0.42898 (− 0.42863), respectively that indicate that these atoms are highly electronegative and tend to donate electrons, while the most electropositive center charge are C13 and S16 atoms with 0.63879 (0.63425), and 0.23081 (0.23448) which means these atoms tend to accept electrons.

### Acidity and basicity

**Scheme 2:**
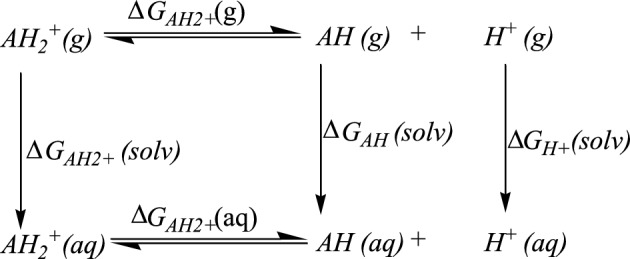
Thermodynamic cycle of the protonated structures *AH*_*2*_^+^ and *AH*^+^ at different phases (gas (g) and aqueous (s)) for *pK*_*a*_ calculation.

**Figure 5:**
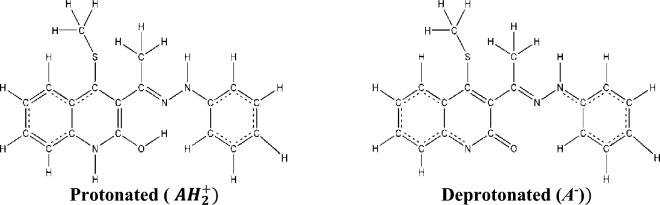
The optimized structure of protonated (${AH}_{2}^{+},$ cation) and deprotonated (*A*^*-*^*,* anion) structures of 4-(methylsulfanyl)-3[(1Z)-1-(2 phenylhydrazinylidene) ethyl] quinoline-2(1H)-one at B3LYP/6-31G(d,p) level.

Investigation of our compound shows it has two protons that are attached to either a nitrogen or oxygen atom. The acidity and basicity of any molecule are required to explain its structure, reactivity, and different chemical properties. Furthermore, learning the acidity constants (*pK*_*a*_) is important for estimating the equilibrium constant (*K*) of different reactions, specifically those involving proton transfers. When determining the *pK*_*a*_ experimentally is difficult, Scheme 2 shows how the thermodynamic free energies cycle can be used to approximate computational methods. The protonated (cation) and deprotonated (anion) structures of the 4-(methylsulfanyl)-3[(1Z)-1-(2 phenylhydrazinylidene) ethyl] quinoline-2(1H)-one are depicted in Fig. 5. *AH*, is usually neutral, denoted ${AH}_{2}^{+}$, typically has a net charge of + 1, for the protonated form, while the corresponding enol/rotamer or keto. The hydroxyl group of the phenylhydrazinylidene ring has been rotated to give the rotamer structure, according to the optimized structure of the deprotonated form. Therefore, the energy of the rotamer will be studied throughout the acidity constant calculation from the protonated form. The deprotonated form denoted ${A}^{-}$, typically has a net charge of − 1.

The equations used for calculating *pK*_*a*_ values are given below (Eqs. (13–16)):

13 $$ pK_{a} = \Delta G_{{AH_{2}^{ + } }} \left( {aq} \right) / 2.303RT $$

14 $$ \Delta G_{{AH_{2}^{ + } }} \left( {aq} \right) = \Delta G_{{AH_{2}^{ + } }} \left( g \right) + \Delta \Delta G_{{AH_{2}^{ + } }} \left( {solv} \right) $$

15 $$ \Delta G_{{AH_{2}^{ + } }} \left( g \right) = G_{AH} \left( g \right) + G_{{H^{ + } }} \left( g \right) - G_{{AH_{2}^{ + } }} \left( g \right) $$

16 $$ \Delta \Delta G_{{AH_{2}^{ + } }} \left( {solv} \right) = \Delta G_{AH} \left( {solv} \right) + \Delta G_{{H^{ + } }} \left( {solv} \right) - \Delta G_{{AH_{2}^{ + } }} \left( {solv} \right) $$

where *G*_*i*_(*g*), *ΔG*_*i*_(*solv*), and *G*_*i*_(*aq*) are the standard free energies of the species "*i*" in the gas phase, the solvation free energy of "*i*", and the free energy change in aqueous phase, respectively. The ${G}_{{H}^{+}}\left(g\right)$ and ${\Delta G}_{{H}^{+}}\left(solv\right)$ terms are − 6.28 kcal/mol^69,70^ and − 265.90 kcal/mol^71,72^, respectively.

To study the strength of the OH and NH bonds, Tables 5 and 6 presented different charges on the N and O atoms for enol, keto, and enol-rotamer structures. Table 7 collects the calculated acidity constant (*pK*_*a*_) for the protonated and deprotonated structures in ethanol at B3LYP and M06-2X levels. From the obtained results, the values show that the correlation between the computational and experimental acidity constants indicates that the B3LYP/6-311++G(2d,2p) level gives the nearest *pK*_*a*_ value to the experimental values.

**Table 7:** Estimated acidity constant (*pK*_*a*_) for the protonated and deprotonated structures in ethanol using B3LYP^a^ and M06-2X^a^ methods.

Equation	B3LYP/6-31G(d,p)	B3LYP^a^	M06-2X^a^
$AH_{2}^{ + } \to AH + H^{ + }$	16.45	12.52	9.32
$AH \, \left( {enol} \right) \, \to \, A^{ - } + \, H^{ + }$	29.09	22.03	21.55
$AH \, \left( {keto} \right) \, \to \, A^{ - } + \, H^{ + }$	27.44	20.29	17.48
$AH \, \left( {rotamer} \right) \, \to \, A^{ - } + \, H^{ + }$	22.79	16.05	16.15

^a^Method/6-311++G(2d,2p)//B3LYP/6-31G(d,p).

The experimental *pK*_*a*_ of 4-(methylsulfanyl)-3[(1Z)-1-(2 phenylhydrazinylidene) ethyl] quinoline-2(1H)-one was 12.7^17^, and the protonation structure estimated *pK*_*a*_ is 17.48, 12.52, and 9.32 at B3LYP/6-31G (d, p), B3LYP/6-311++G (2d, 2p), M06 By comparing the *pK*_*a*_ obtained by deprotonation, the enol form is less acidic than its keto and rotamer structures. This can be credited to some parameters such as the presence or absence of hydrogen bonds, the strength of the OH and NH bonds, and the stability of the resulting conjugate base upon deprotonation.

In the gas phase, the intrinsic basicity can be calculated from the proton affinity (*PA*), which is the negative of the protonation reaction of *AH*.

17 $$ AH + H^{ + } \to AH_{2}^{ + } ,PA = - \Delta H^{ \circ } $$

The higher energy of the keto and rotamer forms indicates that the enol form of 4-(methylsulfanyl)-3[(1Z)-1-(2 phenylhydrazinylidene) ethyl] quinoline-2(1H)-one is stabilized and dominant in nature.

### UV–Vis spectral analysis

The photo physics and photochemistry of any molecule can be determined by its serious ability to act as a dye or sensor. Table S4 collects the first *λ*_*max*_ of the 4-(methylsulfanyl)-3[(1Z)-1-(2 phenylhydrazinylidene) ethyl] quinoline-2(1H)-one using different solvation models (PCM, CPCM, and SMD) and different DFT methods (B3LYP, CAM-B3LYP, PBE, ωB97X-D). The obtained theoretical results are compared against the experimental ones. The TDDFT-PBE/SMD model shows a good agreement with the 1st and 2nd maximum excitation peaks (*E*_*ex*_ is the difference between the computational and experimental 1st maximum and 2nd excitation energies are 0.06/0.07 and 0.09 eV, respectively)^53^.

**Figure 6:**
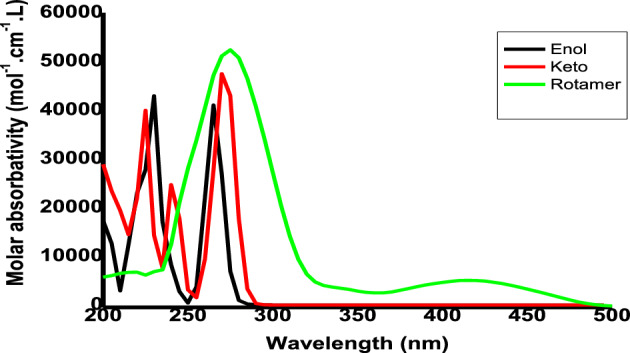
The UV/Vis absorption spectra for different structures keto, enol and rotamer of 4 (methylsulfanyl)-3[(1Z)-1-(2 phenylhydrazinylidene) ethyl] quinoline-2(1H)-one at TD-PBE/6-311+G(d,p) level.

**Figure 7:**
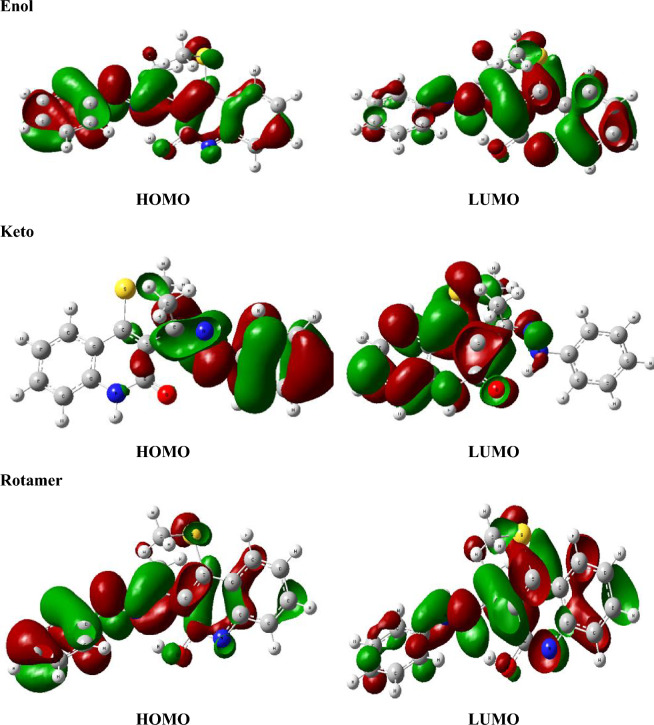
HOMO, LUMO plot of keto, enol and rotamer at TD-PBE/6-311+G(d,p) level.

Table 8 presents the values of *E*_*ex*_ and *f* and the transition configurations of the intense peaks for enol, keto, and rotamer, while Figs. 6 and 7 show the Ultraviolet–Vissible (UV/Vis) absorption spectra of different tautomeric structures of 4 (methylsulfanyl)-3[(1Z)-1-(2 phenylhydrazinylidene) ethyl] quinoline-2(1H)-one and the HOMO and LUMO plots for keto, enol and rotamer at TD-PBE/6-311+G(d,p) level, respectively.

**Table 8:** Excitation energies (*E*_*ex*_, eV), oscillator strengths (*f*), and possible transitions at TD-PBE-SMD,acetonitrile/6-31G(d,p)//B3LYP/6-311+G(d,p) level.

Compound	State	*E*_*ex*_^*a*^	*f* ^*b*^	Assignment ^c^
Enol	1	2.60 (475)	0.3943	H → L (85%)
	4	3.53 (350)	0.3051	H → L + 1 (52%)
	7	3.85 (321)	0.1206	H-3 → L (35%), H-1 → L + 1 (14%), H → L + 2 (11%)
	11	4.30 (288)	0.2004	H-3 → L (11%), H → L + 3 (30%)
Keto	4	3.48 (355)	0.1120	H-1 → L (32%)
	8	4.01 (309)	0.2274	H → L + 2 (30%), H → L + 3 (30%)
	11	4.26 (291)	0.2699	H-7 → L (27%), H → L + 1 (13%), H → L + 3 (18%), H → L + 4(15%)
	12	4.29 (289)	0.1291	H-7 → L (29%), H-5 → L (12%), H-1 → L + 1 (12%), H → L + 5(11%)
Rotamer	4	3.67 (338)	0.1131	H-2 → L (86%)
	7	3.96 (313)	0.1038	H-5 → L (30%), H → L + 4 (24%)
	10	4.06 (305)	0.1239	H-5 → L (12%), H-3 → L (32%), H-1 → L + 1 (15%), H → L + 3 (29%)
	11	4.20 (295)	0.3760	H-2 → L + 1 (11%), H → L + 1 (14%), H → L + 2 (17%), H → L + 3 (29%)
TS1	1	2.21 (561)	0.1731	H → L (100%)
	4	3.46 (358)	0.1851	H → L + 1 (61%), H → L + 3 (12%)
	6	3.83 (324)	0.1165	H-3 → L (36%), H → L + 3 (10%), H → L + 4 (16%)
	11	4.20 (295)	0.2670	H-6 → L (16%), H → L + 3 (25%)
	12	4.24 (292)	0.1374	H-6 → L (31%), H-2 → L + 1 (26%)
TS2	1	2.25 (550)	0.2036	H → L (100%)
	4	3.48 (356)	0.1963	H → L + 1 (63%)
	6	3.81 (325)	0.1061	H-5 → L (15%), H-3 → L (39%)
	11	4.23 (293)	0.2713	H → L + 1 (13%), H → L + 2 (14%), H → L + 3 (26%), H → L + 4 (12%)
	12	4.27 (290)	0.1175	H-6 → L (41%), H-2 → L + 1 (27%), H → L + 3 (12%)

^a^Values in parentheses are given in nm.

^b^Oscillator strengths (*f* > 0.1).

^c^Only contributions above 10% are shown. H and L represent HOMO and LUMO, respectively.

The presence of HBs in chemical compounds can contribute to their chemical stability^73,74^ and is considered a strong reason for the strength, shifting, and broadening of the absorption peaks. Figure 6 depicts the keto structure with the strongest HB and a large blue shift (until 280 nm), followed by the enol structure (until 300 nm), and finally the lower shift in the rotamer (until 500 nm). The blue shift and hyperchromic effect for keto and enol forms can be returns to the presence of HBs^17^.

As shown in Table 4, the lower *Eg* of the keto, enol, and rotamer have maximums in the electronic absorption spectra of keto relative to enol and rotamer, which are bathochromically shifted by 17 and 66 nm, respectively. In the UV–Vis spectra, the peaks of keto, enol, and rotamer are extended over 348–438 nm, 272–500 nm, and 280–479 nm, respectively. The strong electronic absorption of keto is assigned to the HOMO^−3^ to LUMO and HOMO^−2^ to LUMO^+1^ transitions. The maximum absorption peak for enol, which appears at a lower wavelength than keto, is attributed to HOMO^−2^ to LUMO^+1^ and HOMO^−1^ to LUMO^+1^ transitions. The broad electronic absorption of rotamer can be attributed to the transition of HOMO^−1^ to LUMO^+2^.

**Figure 8:**
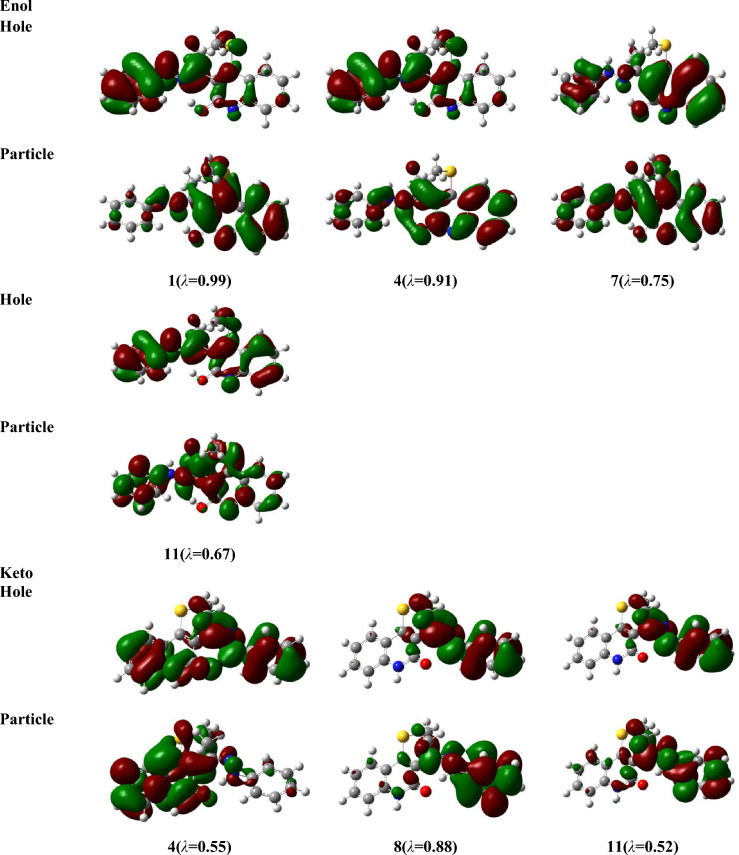

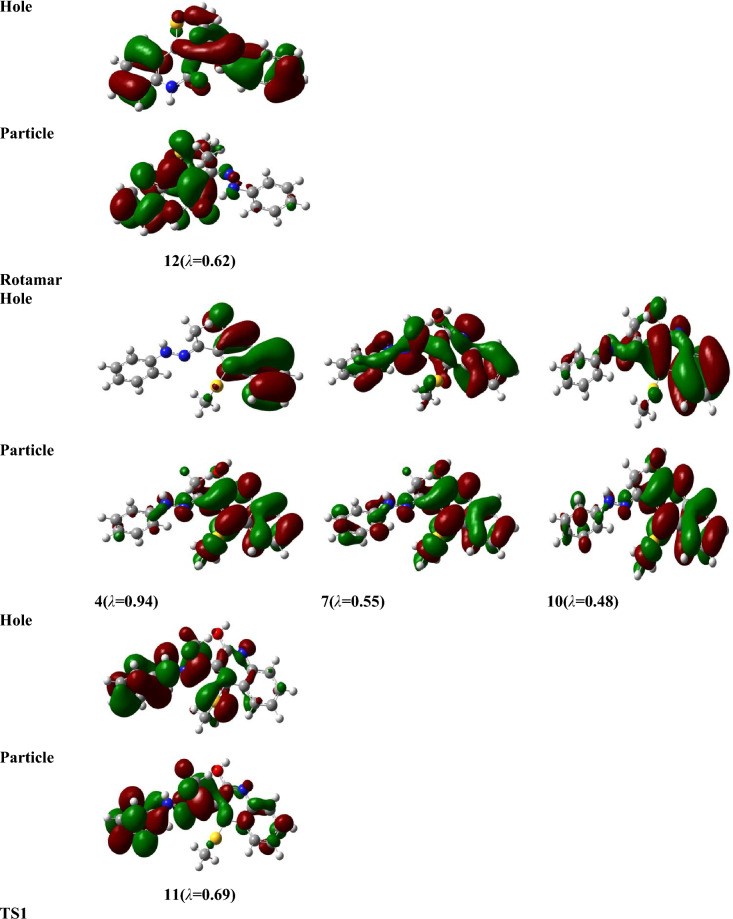

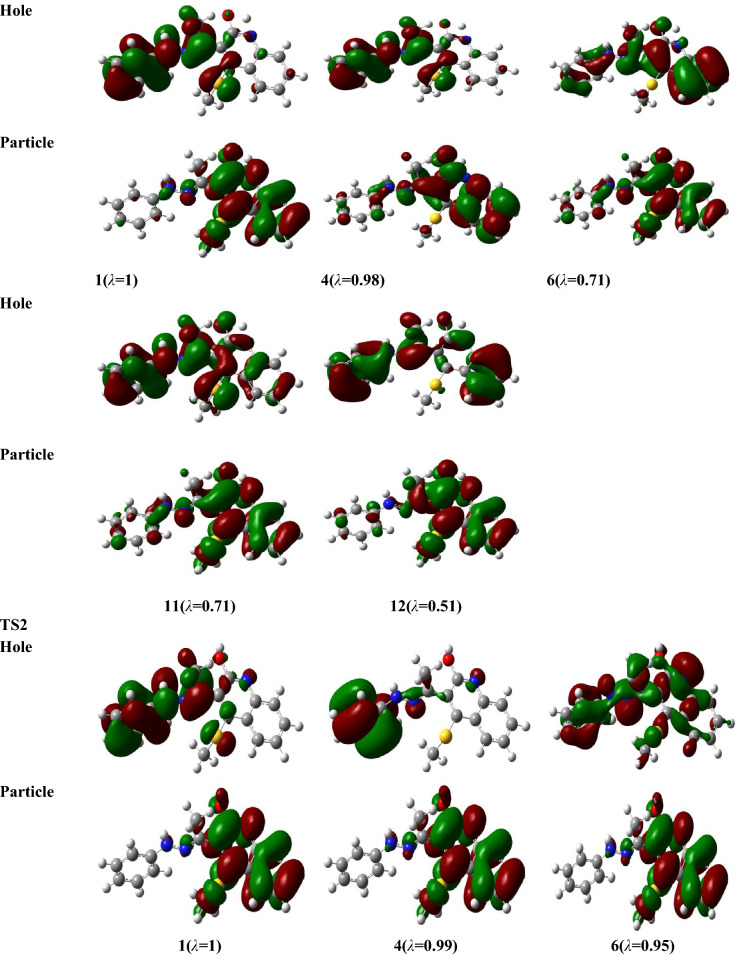

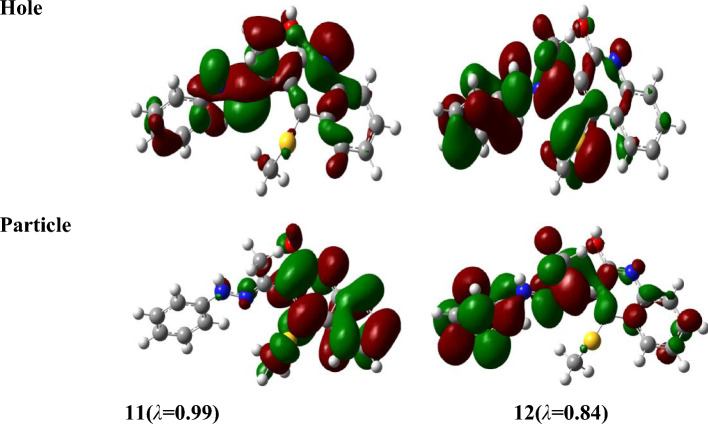
Natural transition orbitals (NTOs) of keto, enol, rotamar and transition states for the excitation with significant oscillation strengths at the PBE/6-311+G(d,p) level with solvent effects of acetonitrile through SMD. The occupied (holes) and unoccupied (electrons) NTO pairs are only that have contribution more than 50% to each excited state (λ is eigenvalues of the pairs).

To analyze the nature of UV–Vis absorption, Fig. 8 shows the NTOs of keto, enol, rotamar and transition states for the high-intensity excited states at the PBE/6-311+G(d,p) level with solvent effects of acetonitrile through SMD. In Fig. 8, the occupied NTOs are referred to “hole”, while the unoccupied NTOs are the “particles” transition orbitals. NTOs can provide a simple description of the excited state rather than the canonical orbitals. For the investigated structures, the dominant transitions are expected to be π − π* and n − π* excitations which makes the analysis of excitations are difficult. However, from Fig. 8, the holes of NTOs can reproduce the bands given in Fig. 6 and Table 8 and the holes are seem to delocalize on the whole molecular structure while the particles NTOs are mainly delocalized on benzene rings which enhances the π–π* excitation.

## Conclusions

The density functional methods (DFT) were used to investigate the tautomer and related rotamers of 4 (methylsulfanyl)-3 [(1Z)-1-(2 phenylhydrazinylidene) ethyl] quinoline-2(1H)-one. B3LYP and M06-2× connected with 6-31G (d, p) and 6-311++G (2d, 2p) basis sets have been used for analysis of different structural properties, stability, and aromaticity. The obtained thermo-kinetic results show a relative stability for keto form compared to other forms and high barriers required for enol formation in gas phase and ethanol under the applied temperature range. Using Eckert tunneling correction indicates a great contribution in keto-enol conversion compered to enol-rotomar conversion. Different sites for the nucleophilic and electrophilic reactions were allocated using Fukui functions. UV absorption spectra in ethanol and gas phases were investigated using the time-dependent density functional theory (TD-DFT) methods B3LYP, PBE, PBE0, CAM-B3LYP, M06-2X, b97X-D, and CIS. The TDDFT-PBE/SMD approach shows good harmony with the 1st and 2nd maximum excitation peaks, and the transitions are π–π* transitions. The remarkable chemical shift of a proton at 14.76 and 19.40 ppm in the nuclear magnetic resonance spectrum has been attributed to the existence of low-barrier hydrogen bonds (LBHBs) for the enol and keto forms, respectively.

## Supplementary Information

Supplementary Information.

## Data Availability

All data generated through this study are included in this manuscript and the Supporting Information file.
