# Supplementary material for: First-principles density functional theoretical study on the structures, reactivity and spectroscopic properties of (NH) and (OH) Tautomer's of 4-(methylsulfanyl)-3[(1Z)-1-(2-phenylhydrazinylidene) ethyl] quinoline-2(1H)-one
Source: Sci Rep. 2023 Jun 1;13:8909. doi: 10.1038/s41598-023-35933-8 (PMC10235042; doi:10.1038/s41598-023-35933-8)
Supplement: Supplementary file 1 — Supplementary Information. [file 41598_2023_35933_MOESM1_ESM.docx]

**[First-Principles Density Functional Theoretical Study](https://www.researchgate.net/publication/352815320_First-Principles_Kinetic_Studies_of_Unimolecular_Pyrolysis_of_Isopropyl_Esters_as_Biodiesel_Surrogates?_sg%5B0%5D=WUqvw-TOUbpAE_YXe3wVl07mztLg3dmI_B0btLBMkil7jd93iziWN2hPiV326v3sW7y0gQr1tkX4XRx4KB9LY_iJXIhj5wBPC5vxN07A.L62DlTooqvtlD3ArYfQZ4kz4N1F44bdhTgw7MZuSNyHNSnw9mdmmhlwBW4eB7q8c8_QxbSiiskDB07C5f4JHvw) on the Structures, Reactivity and Spectroscopic Properties of (NH) and (OH) Tautomer's of 4-(methylsulfanyl)-3[(1Z)-1-(2-phenylhydrazinylidene) ethyl] quinoline-2(1H)-one**

**Shimaa Abdel Halim^a,^ * Mohamed A. Abdel-Rahman ^b,^** **^*^**

*^a^* *Chemistry Department, Faculty of Education, Ain Shams University, Roxy 11711, Cairo, Egypt*

*^b^ Chemistry Department, Faculty of Science, Suez University, Suez, 43518, Egypt*

****Corresponding Author***

*E-mail*:  [Shimaaquantum@ymail.com](mailto:%20Shimaaquantum@ymail.com) (Shimaa Abdel Halim). **Mohamed.Abdel-Rahman@sci.suezuni.edu.eg** (Mohamed A. Abdel-Rahman)

| 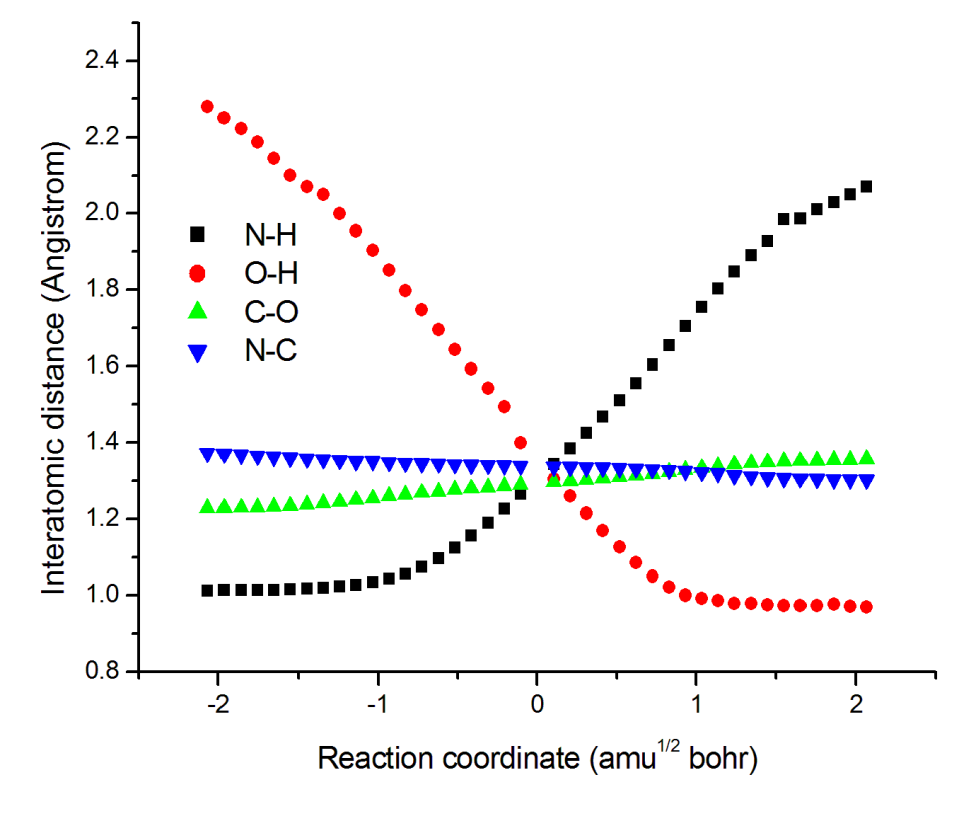 |
| --- |
| Fig S1. Change of bond lengths (angstroms) along reaction coordinates for tautomarization at B3LYP/6-31G(d,p) level. |
| 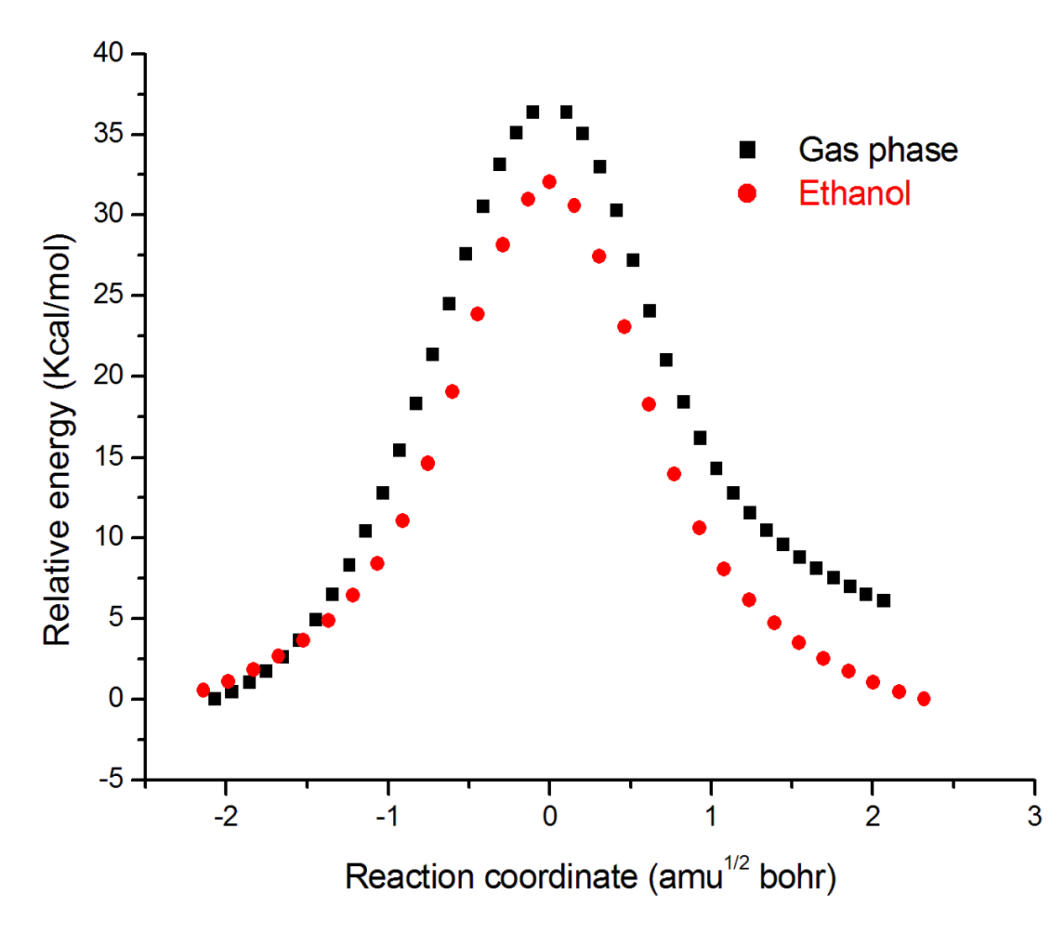 |
| Fig S2. Potential energy profiles during enol-keto reaction in gas phase and ethanol at B3LYP/6-31G(d,p) level. |

| 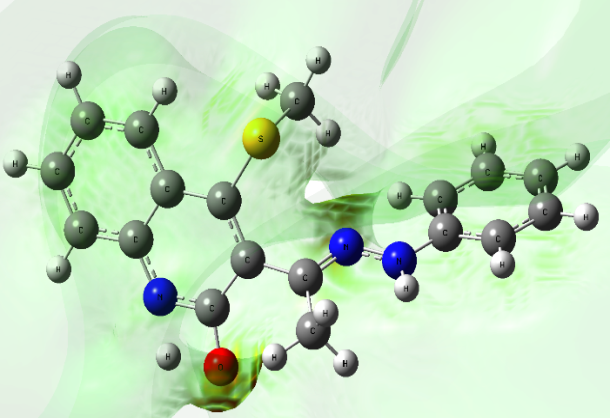 | 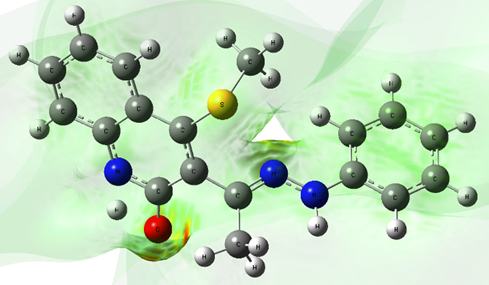 |
| --- | --- |
| A | B |
| Fig. S3. Molecular ESP surfaces of the TS in gas phase (A) and ethanol (B) phase at M062X/6 311++G(2d,2p). | |

Fig. S4. NBO atomic charges of the TS in gas phase at M062X/6 311++G(2d,2p).

Fig. S5. NBO atomic charges of the TS in ethanol phase at M062X/6 311++G(2d,2p).

Table S1 optimized geometries of Enol, Keto, Rotomar and different transition states at B3LYP/6-31G(d,p) level.

| 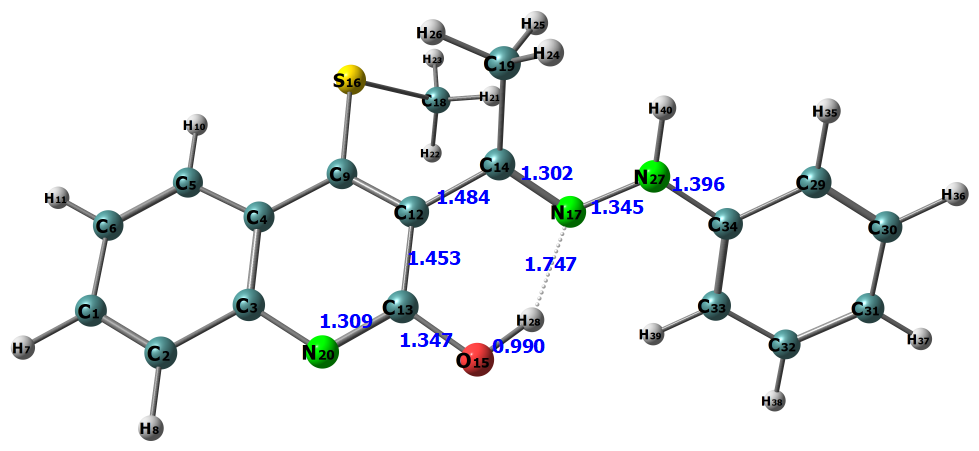  Enol in gas phase  6 5.613865000 -1.490130000 0.193141000  6 4.425876000 -2.139457000 -0.056426000  6 3.205765000 -1.417649000 -0.115066000  6 3.224709000 0.002314000 0.071763000  6 4.463988000 0.636562000 0.353469000  6 5.631945000 -0.091992000 0.406265000  1 6.541743000 -2.053127000 0.237227000  1 4.377404000 -3.212741000 -0.206147000  6 1.974604000 0.687766000 -0.060249000  1 4.477505000 1.707504000 0.519652000  1 6.572001000 0.409492000 0.615564000  6 0.820184000 -0.020055000 -0.382848000  6 0.924851000 -1.471450000 -0.386492000  6 -0.458976000 0.622215000 -0.777426000  8 -0.175301000 -2.233932000 -0.500692000  16 1.923793000 2.465062000 0.236066000  7 -1.547434000 0.067595000 -0.329168000  6 0.857032000 2.526333000 1.729330000  6 -0.498756000 1.746830000 -1.787297000  7 2.057211000 -2.120675000 -0.302999000  1 -0.132052000 2.109630000 1.533392000  1 1.329783000 1.994058000 2.556209000  1 0.762149000 3.583448000 1.987624000  1 -1.120660000 1.441516000 -2.641173000  1 -0.926441000 2.668147000 -1.372509000  1 0.493896000 1.987779000 -2.158399000  7 -2.750806000 0.526579000 -0.722618000  1 -0.954620000 -1.647594000 -0.401006000  6 -5.152334000 0.588711000 -0.596518000  6 -6.356432000 0.117730000 -0.080830000  6 -6.369872000 -0.910701000 0.863315000  6 -5.158201000 -1.459874000 1.286817000  6 -3.943623000 -0.998253000 0.783738000  6 -3.937360000 0.031962000 -0.167838000  1 -5.150189000 1.385896000 -1.336356000  1 -7.288483000 0.558447000 -0.422233000  1 -7.309716000 -1.277592000 1.262859000  1 -5.152281000 -2.258179000 2.023266000  1 -3.006973000 -1.420860000 1.125472000  1 -2.807736000 1.374985000 -1.272545000  Zero-point correction= 0.316125 (Hartree/Particle)  Thermal correction to Energy= 0.336721  Thermal correction to Enthalpy= 0.337665  Thermal correction to Gibbs Free Energy= 0.265376  Sum of electronic and zero-point Energies= -1333.515791  Sum of electronic and thermal Energies= -1333.495195  Sum of electronic and thermal Enthalpies= -1333.494250  Sum of electronic and thermal Free Energies= -1333.566540 |
| --- |
| Enol in gas phase  6 5.597225000 -1.589018000 0.084862000  6 4.379606000 -2.205340000 -0.110574000  6 3.178832000 -1.451963000 -0.113560000  6 3.237999000 -0.035948000 0.075481000  6 4.505366000 0.562323000 0.305239000  6 5.658871000 -0.194204000 0.301187000  1 6.511051000 -2.176354000 0.084010000  1 4.305584000 -3.278069000 -0.261368000  6 1.999779000 0.690971000 -0.001333000  1 4.564915000 1.630370000 0.480639000  1 6.618643000 0.285031000 0.470054000  6 0.817534000 0.016908000 -0.301963000  6 0.887856000 -1.434336000 -0.302396000  6 -0.450736000 0.679463000 -0.695132000  8 -0.237648000 -2.170860000 -0.376377000  16 2.056256000 2.456002000 0.316067000  7 -1.545262000 0.108896000 -0.280630000  6 0.710481000 2.700573000 1.536756000  6 -0.470604000 1.836846000 -1.660872000  7 1.999181000 -2.124126000 -0.252934000  1 -0.282999000 2.610135000 1.097436000  1 0.823074000 2.001582000 2.367423000  1 0.843466000 3.720267000 1.907587000  1 -1.070612000 1.559636000 -2.538570000  1 -0.926770000 2.734899000 -1.226127000  1 0.527739000 2.095937000 -2.005460000  7 -2.743526000 0.580379000 -0.668326000  1 -1.005573000 -1.553151000 -0.284610000  6 -5.145181000 0.600932000 -0.599136000  6 -6.353476000 0.085707000 -0.137125000  6 -6.374993000 -0.991701000 0.753407000  6 -5.164645000 -1.546311000 1.176758000  6 -3.944787000 -1.042635000 0.725878000  6 -3.930117000 0.038422000 -0.170769000  1 -5.133502000 1.437069000 -1.293906000  1 -7.284044000 0.531212000 -0.477895000  1 -7.318975000 -1.390942000 1.111545000  1 -5.163689000 -2.381737000 1.871773000  1 -3.012296000 -1.475644000 1.068473000  1 -2.801722000 1.440433000 -1.204080000  Zero-point correction= 0.315918 (Hartree/Particle)  Thermal correction to Energy= 0.336295  Thermal correction to Enthalpy= 0.337239  Thermal correction to Gibbs Free Energy= 0.265773  Sum of electronic and zero-point Energies= -1333.543532  Sum of electronic and thermal Energies= -1333.523155  Sum of electronic and thermal Enthalpies= -1333.522210  Sum of electronic and thermal Free Energies= -1333.593676 |
| Keto in gas phase  6 -5.341863000 -1.496621000 -0.689972000  6 -4.246045000 -2.096873000 -0.092051000  6 -3.071444000 -1.355880000 0.116473000  6 -2.987889000 0.003870000 -0.270831000  6 -4.117316000 0.575271000 -0.895602000  6 -5.277108000 -0.155316000 -1.096402000  1 -6.248366000 -2.072148000 -0.849739000  1 -4.280606000 -3.138107000 0.216185000  6 -1.754442000 0.729124000 0.021463000  1 -4.068428000 1.605493000 -1.226936000  1 -6.132239000 0.310396000 -1.574900000  6 -0.709169000 0.120371000 0.683665000  6 -0.751969000 -1.328833000 0.927875000  6 0.456742000 0.870297000 1.259550000  6 3.885919000 -1.561711000 -0.826983000  6 5.228211000 -1.718126000 -1.162599000  6 6.151249000 -0.706217000 -0.890753000  6 5.708517000 0.467266000 -0.276590000  6 4.367898000 0.640037000 0.061072000  6 3.444499000 -0.380416000 -0.210220000  1 3.173142000 -2.354991000 -1.038306000  1 5.553448000 -2.640432000 -1.635923000  1 7.197376000 -0.830474000 -1.152635000  1 6.415158000 1.262914000 -0.056094000  1 4.023040000 1.546724000 0.542490000  8 0.206239000 -1.996544000 1.332409000  16 -1.720173000 2.433203000 -0.516807000  7 1.704039000 0.709911000 0.972404000  7 2.075082000 -0.240294000 0.067323000  6 -0.008697000 2.694961000 -1.120290000  6 0.149747000 1.882362000 2.339325000  7 -1.960610000 -1.949959000 0.675961000  1 0.666610000 3.003276000 -0.324206000  1 0.367256000 1.785982000 -1.591447000  1 -0.091829000 3.486867000 -1.867676000  1 -0.222667000 1.375247000 3.238109000  1 1.065402000 2.416077000 2.601591000  1 -0.617335000 2.600453000 2.033404000  1 1.553497000 -1.113921000 0.139968000  1 -1.971214000 -2.941256000 0.883130000  Zero-point correction= 0.316770 (Hartree/Particle)  Thermal correction to Energy= 0.337270  Thermal correction to Enthalpy= 0.338214  Thermal correction to Gibbs Free Energy= 0.266657  Sum of electronic and zero-point Energies= -1333.525457  Sum of electronic and thermal Energies= -1333.504958  Sum of electronic and thermal Enthalpies= -1333.504014  Sum of electronic and thermal Free Energies= -1333.575571 |
| Keto in ethanol  6 -5.131916000 -1.655544000 -0.799587000  6 -4.118959000 -2.118291000 0.023111000  6 -2.988149000 -1.316230000 0.255796000  6 -2.865115000 -0.036025000 -0.339887000  6 -3.911899000 0.394582000 -1.186364000  6 -5.028093000 -0.394233000 -1.409255000  1 -6.004998000 -2.276220000 -0.977289000  1 -4.180371000 -3.094743000 0.494694000  6 -1.672857000 0.749944000 -0.038308000  1 -3.836534000 1.357615000 -1.677152000  1 -5.818385000 -0.038407000 -2.062531000  6 -0.701530000 0.255256000 0.802107000  6 -0.825170000 -1.090678000 1.381209000  6 0.461299000 1.037929000 1.331858000  6 3.541507000 -1.346910000 -1.270151000  6 4.845851000 -1.676605000 -1.629359000  6 5.935670000 -1.044336000 -1.022986000  6 5.695874000 -0.073931000 -0.046457000  6 4.396862000 0.267934000 0.327740000  6 3.303959000 -0.369055000 -0.286148000  1 2.698998000 -1.842669000 -1.746228000  1 5.008973000 -2.435275000 -2.390258000  1 6.951346000 -1.304511000 -1.305220000  1 6.530732000 0.426691000 0.437236000  1 4.219492000 1.018923000 1.088259000  8 0.020017000 -1.597711000 2.135564000  16 -1.623311000 2.378804000 -0.778348000  7 1.692241000 0.842481000 0.999167000  7 1.986849000 -0.063108000 0.039559000  6 0.125705000 2.690814000 -1.221630000  6 0.177565000 2.053598000 2.404937000  7 -1.972091000 -1.778986000 1.063749000  1 0.705078000 3.081492000 -0.386813000  1 0.587255000 1.787088000 -1.622336000  1 0.077813000 3.444498000 -2.012002000  1 -0.247113000 1.564570000 3.291223000  1 1.098515000 2.565726000 2.695226000  1 -0.553199000 2.798883000 2.070640000  1 1.245750000 -0.607353000 -0.392662000  1 -2.063136000 -2.697504000 1.487187000  Zero-point correction= 0.316162 (Hartree/Particle)  Thermal correction to Energy= 0.336881  Thermal correction to Enthalpy= 0.337826  Thermal correction to Gibbs Free Energy= 0.265120  Sum of electronic and zero-point Energies= -1333.553816  Sum of electronic and thermal Energies= -1333.533096  Sum of electronic and thermal Enthalpies= -1333.532152  Sum of electronic and thermal Free Energies= -1333.604858 |
| Rotomar in gas phase  6 5.531290000 -0.808578000 -0.958712000  6 4.845836000 0.365661000 -1.181932000  6 3.537142000 0.542109000 -0.670083000  6 2.925344000 -0.510989000 0.081106000  6 3.663429000 -1.702434000 0.298712000  6 4.935861000 -1.849306000 -0.211573000  1 6.534617000 -0.935258000 -1.354900000  1 5.279593000 1.183518000 -1.747597000  6 1.590886000 -0.290842000 0.570193000  1 3.207110000 -2.493370000 0.883036000  1 5.485265000 -2.769209000 -0.035788000  6 0.950333000 0.906673000 0.299256000  6 1.694524000 1.884443000 -0.442727000  6 -0.435990000 1.244937000 0.724602000  8 1.074314000 3.067492000 -0.675744000  16 0.802200000 -1.528133000 1.617062000  7 -1.393872000 0.585767000 0.164717000  6 -0.080002000 -2.538425000 0.364356000  6 -0.639571000 2.333272000 1.752503000  7 2.903489000 1.728999000 -0.909401000  1 -0.835754000 -1.930509000 -0.133126000  1 0.622029000 -2.955996000 -0.360127000  1 -0.557893000 -3.352967000 0.913625000  1 -1.378714000 3.068995000 1.411025000  1 -0.991485000 1.911690000 2.704585000  1 0.291163000 2.865193000 1.949067000  7 -2.665652000 0.852271000 0.531277000  6 -5.019155000 0.340069000 0.504973000  6 -6.117458000 -0.292727000 -0.070618000  6 -5.964867000 -1.084599000 -1.210411000  6 -4.693687000 -1.227517000 -1.770803000  6 -3.585033000 -0.595568000 -1.212276000  6 -3.742338000 0.191191000 -0.060335000  1 -5.146092000 0.948670000 1.397462000  1 -7.097907000 -0.167774000 0.380082000  1 -6.822369000 -1.578580000 -1.655963000  1 -4.559955000 -1.834121000 -2.662239000  1 -2.602586000 -0.689408000 -1.657889000  1 -2.852242000 1.427872000 1.343690000  1 1.708492000 3.597969000 -1.185538000  Zero-point correction= 0.315634 (Hartree/Particle)  Thermal correction to Energy= 0.336602  Thermal correction to Enthalpy= 0.337546  Thermal correction to Gibbs Free Energy= 0.264226  Sum of electronic and zero-point Energies= -1333.517811  Sum of electronic and thermal Energies= -1333.496843  Sum of electronic and thermal Enthalpies= -1333.495899  Sum of electronic and thermal Free Energies= -1333.569220 |
| Rotomar in ethanol  6 5.525731000 -0.740124000 -1.014300000  6 4.816608000 0.424005000 -1.223049000  6 3.512651000 0.577870000 -0.691035000  6 2.930934000 -0.486169000 0.067619000  6 3.690976000 -1.666968000 0.268189000  6 4.958510000 -1.792944000 -0.262250000  1 6.524876000 -0.848458000 -1.426577000  1 5.234891000 1.246885000 -1.794690000  6 1.601306000 -0.286603000 0.580043000  1 3.260623000 -2.472893000 0.852044000  1 5.524276000 -2.705460000 -0.099193000  6 0.937808000 0.899388000 0.320811000  6 1.649351000 1.892090000 -0.435984000  6 -0.447541000 1.217305000 0.771091000  8 0.989073000 3.051684000 -0.662259000  16 0.847515000 -1.536088000 1.639855000  7 -1.400838000 0.568325000 0.188876000  6 0.044769000 -2.624542000 0.400478000  6 -0.651083000 2.269437000 1.832605000  7 2.854497000 1.755569000 -0.921416000  1 -0.740840000 -2.078859000 -0.124571000  1 0.776812000 -3.019850000 -0.306938000  1 -0.395011000 -3.453068000 0.961895000  1 -1.360235000 3.039021000 1.501353000  1 -1.057219000 1.821343000 2.750037000  1 0.288834000 2.759419000 2.090896000  7 -2.676442000 0.815646000 0.564658000  6 -5.033340000 0.343457000 0.482769000  6 -6.129435000 -0.271032000 -0.117229000  6 -5.968097000 -1.063619000 -1.257864000  6 -4.687491000 -1.225984000 -1.792645000  6 -3.579019000 -0.613728000 -1.208392000  6 -3.744831000 0.175089000 -0.056427000  1 -5.164792000 0.954579000 1.372313000  1 -7.116867000 -0.130630000 0.314442000  1 -6.824766000 -1.542532000 -1.722288000  1 -4.544045000 -1.832948000 -2.682830000  1 -2.592147000 -0.731287000 -1.639570000  1 -2.873924000 1.389229000 1.378213000  1 1.579352000 3.631107000 -1.175554000  Zero-point correction= 0.315227 (Hartree/Particle)  Thermal correction to Energy= 0.336138  Thermal correction to Enthalpy= 0.337082  Thermal correction to Gibbs Free Energy= 0.263953  Sum of electronic and zero-point Energies= -1333.543407  Sum of electronic and thermal Energies= -1333.522497  Sum of electronic and thermal Enthalpies= -1333.521552  Sum of electronic and thermal Free Energies= -1333.594681 |
| TS1 (rotomar- Enol) in gas phase  6 5.511690000 -0.784859000 -1.027086000  6 4.807306000 0.376280000 -1.252444000  6 3.509995000 0.548626000 -0.706447000  6 2.930765000 -0.498462000 0.079763000  6 3.688657000 -1.678351000 0.299713000  6 4.947372000 -1.817851000 -0.243155000  1 6.504909000 -0.909825000 -1.448226000  1 5.212633000 1.189712000 -1.844704000  6 1.609162000 -0.282904000 0.593567000  1 3.258323000 -2.464318000 0.909923000  1 5.512784000 -2.727887000 -0.065868000  6 0.958317000 0.907824000 0.309107000  6 1.671654000 1.889703000 -0.458577000  6 -0.430837000 1.224475000 0.747810000  8 1.024256000 3.066769000 -0.770694000  16 0.842009000 -1.511733000 1.666573000  7 -1.387915000 0.586174000 0.165644000  6 -0.056496000 -2.536750000 0.437143000  6 -0.644612000 2.282190000 1.807203000  7 2.867999000 1.729180000 -0.948684000  1 -0.816054000 -1.934235000 -0.061494000  1 0.637142000 -2.964713000 -0.289245000  1 -0.530186000 -3.343112000 1.001844000  1 -1.299724000 3.086262000 1.446035000  1 -1.108288000 1.852207000 2.705874000  1 0.302030000 2.722864000 2.127114000  7 -2.661935000 0.849745000 0.523759000  6 -5.012386000 0.322525000 0.485157000  6 -6.104537000 -0.314325000 -0.097999000  6 -5.941551000 -1.101067000 -1.239725000  6 -4.666891000 -1.235162000 -1.794477000  6 -3.564401000 -0.599331000 -1.228194000  6 -3.732734000 0.182509000 -0.074884000  1 -5.147130000 0.927457000 1.379060000  1 -7.087874000 -0.196503000 0.348225000  1 -6.794043000 -1.598194000 -1.691327000  1 -4.525513000 -1.837806000 -2.687337000  1 -2.579086000 -0.686409000 -1.668832000  1 -2.857121000 1.411322000 1.344141000  1 1.041129000 3.660629000 -0.008685000  Zero-point correction= 0.313940 (Hartree/Particle)  Thermal correction to Energy= 0.334733  Thermal correction to Enthalpy= 0.335677  Thermal correction to Gibbs Free Energy= 0.262589  Sum of electronic and zero-point Energies= -1333.501997  Sum of electronic and thermal Energies= -1333.481204  Sum of electronic and thermal Enthalpies= -1333.480260  Sum of electronic and thermal Free Energies= -1333.553348 |
| TS1 (rotomar- Enol) in ethanol  6 5.513123000 -0.739165000 -1.047882000  6 4.796325000 0.417383000 -1.264014000  6 3.499129000 0.574098000 -0.713468000  6 2.931614000 -0.480754000 0.069866000  6 3.700799000 -1.655863000 0.277765000  6 4.959830000 -1.782161000 -0.269726000  1 6.506346000 -0.851705000 -1.472777000  1 5.200773000 1.233670000 -1.854589000  6 1.609933000 -0.279986000 0.592604000  1 3.283615000 -2.455024000 0.879986000  1 5.532650000 -2.689285000 -0.100905000  6 0.944428000 0.904991000 0.318381000  6 1.641644000 1.893524000 -0.452057000  6 -0.443070000 1.216862000 0.769871000  8 0.973092000 3.058002000 -0.763276000  16 0.866029000 -1.519056000 1.671580000  7 -1.393117000 0.564827000 0.186943000  6 0.051756000 -2.618948000 0.449570000  6 -0.653219000 2.270066000 1.828976000  7 2.838225000 1.748046000 -0.951330000  1 -0.736322000 -2.076851000 -0.075493000  1 0.778318000 -3.022533000 -0.258804000  1 -0.385511000 -3.440739000 1.022682000  1 -1.342624000 3.051595000 1.483820000  1 -1.086721000 1.828548000 2.736918000  1 0.289058000 2.743108000 2.112736000  7 -2.670750000 0.812955000 0.556561000  6 -5.026729000 0.337866000 0.469768000  6 -6.120195000 -0.281630000 -0.129837000  6 -5.954138000 -1.082109000 -1.264277000  6 -4.671470000 -1.247281000 -1.793218000  6 -3.565514000 -0.630172000 -1.209270000  6 -3.736113000 0.166754000 -0.063599000  1 -5.161859000 0.955217000 1.354451000  1 -7.109304000 -0.138877000 0.297215000  1 -6.808784000 -1.564857000 -1.728453000  1 -4.524358000 -1.860393000 -2.678573000  1 -2.577001000 -0.749933000 -1.636056000  1 -2.871971000 1.391798000 1.365438000  1 1.083191000 3.705504000 -0.048598000  Zero-point correction= 0.313891 (Hartree/Particle)  Thermal correction to Energy= 0.334558  Thermal correction to Enthalpy= 0.335502  Thermal correction to Gibbs Free Energy= 0.262840  Sum of electronic and zero-point Energies= -1333.532923  Sum of electronic and thermal Energies= -1333.512255  Sum of electronic and thermal Enthalpies= -1333.511311  Sum of electronic and thermal Free Energies= -1333.583974 |
| TS2 (Enol-Keto) in gas phase  6 5.521076000 -1.021516000 -0.785132000  6 4.933149000 0.202130000 -1.037224000  6 3.618484000 0.451019000 -0.592752000  6 2.881285000 -0.552831000 0.113859000  6 3.529060000 -1.788500000 0.357510000  6 4.817396000 -2.021101000 -0.083270000  1 6.533884000 -1.212783000 -1.127232000  1 5.459123000 0.987280000 -1.570112000  6 1.525949000 -0.257791000 0.538364000  1 2.991173000 -2.549673000 0.910401000  1 5.291125000 -2.977261000 0.114991000  6 0.933138000 0.967998000 0.260102000  6 1.765570000 1.927253000 -0.411370000  6 -0.453917000 1.376350000 0.582763000  8 1.534596000 3.151121000 -0.761087000  16 0.659629000 -1.468596000 1.556369000  7 -1.407472000 0.618324000 0.146789000  6 -0.166765000 -2.498135000 0.282735000  6 -0.674872000 2.646142000 1.370905000  7 3.013016000 1.652499000 -0.809764000  1 -0.910453000 -1.900598000 -0.243229000  1 0.562737000 -2.918223000 -0.412721000  1 -0.656198000 -3.310800000 0.824664000  1 -1.457291000 3.263816000 0.912606000  1 -0.979164000 2.420580000 2.402864000  1 0.233364000 3.246523000 1.407604000  7 -2.678845000 0.942422000 0.452356000  1 2.796515000 2.887021000 -1.170938000  6 -5.029816000 0.420019000 0.498377000  6 -6.130146000 -0.268744000 -0.004360000  6 -5.983664000 -1.164503000 -1.065469000  6 -4.717862000 -1.353211000 -1.624250000  6 -3.608137000 -0.664294000 -1.140087000  6 -3.758203000 0.223911000 -0.063744000  1 -5.150580000 1.106858000 1.332904000  1 -7.106875000 -0.106840000 0.442461000  1 -6.842787000 -1.702136000 -1.453601000  1 -4.590200000 -2.038192000 -2.457819000  1 -2.631572000 -0.788543000 -1.591286000  1 -2.870871000 1.643661000 1.157817000  Zero-point correction= 0.310870 (Hartree/Particle)  Thermal correction to Energy= 0.331593  Thermal correction to Enthalpy= 0.332537  Thermal correction to Gibbs Free Energy= 0.259765  Sum of electronic and zero-point Energies= -1333.468932  Sum of electronic and thermal Energies= -1333.448210  Sum of electronic and thermal Enthalpies= -1333.447266  Sum of electronic and thermal Free Energies= -1333.520038 |
| TS2 (Enol-Keto) in ethanol  6 5.524189000 -0.899160000 -0.877833000  6 4.890577000 0.304665000 -1.116167000  6 3.581644000 0.510854000 -0.634212000  6 2.896824000 -0.511624000 0.097910000  6 3.590296000 -1.726637000 0.325240000  6 4.872136000 -1.918651000 -0.153086000  1 6.532621000 -1.060012000 -1.247640000  1 5.377519000 1.103533000 -1.666373000  6 1.548216000 -0.254841000 0.562749000  1 3.098287000 -2.509486000 0.890650000  1 5.381958000 -2.859056000 0.032225000  6 0.912365000 0.952400000 0.299554000  6 1.691843000 1.922735000 -0.409684000  6 -0.477279000 1.329292000 0.658816000  8 1.397711000 3.134726000 -0.787208000  16 0.736389000 -1.484469000 1.603232000  7 -1.422581000 0.578606000 0.191746000  6 0.034167000 -2.623150000 0.348847000  6 -0.707242000 2.561778000 1.498209000  7 2.931355000 1.692043000 -0.841337000  1 -0.739576000 -2.112622000 -0.226498000  1 0.812036000 -3.012318000 -0.311440000  1 -0.409773000 -3.451834000 0.906710000  1 -1.398119000 3.259361000 1.006969000  1 -1.146022000 2.291666000 2.468761000  1 0.225684000 3.091769000 1.692130000  7 -2.698370000 0.885431000 0.500804000  1 2.647624000 2.913709000 -1.228826000  6 -5.067932000 0.504788000 0.383972000  6 -6.168008000 -0.160559000 -0.150627000  6 -6.000108000 -1.145349000 -1.128973000  6 -4.709273000 -1.449406000 -1.568809000  6 -3.596288000 -0.788737000 -1.049338000  6 -3.769594000 0.193393000 -0.059054000  1 -5.203933000 1.266027000 1.148237000  1 -7.163676000 0.093386000 0.203005000  1 -6.860002000 -1.662964000 -1.543153000  1 -4.561460000 -2.207247000 -2.333736000  1 -2.600934000 -1.017747000 -1.410317000  1 -2.908159000 1.644236000 1.141684000  Zero-point correction= 0.310101 (Hartree/Particle)  Thermal correction to Energy= 0.330994  Thermal correction to Enthalpy= 0.331938  Thermal correction to Gibbs Free Energy= 0.257740  Sum of electronic and zero-point Energies= -1333.493389  Sum of electronic and thermal Energies= -1333.472496  Sum of electronic and thermal Enthalpies= -1333.471552  Sum of electronic and thermal Free Energies= -1333.545751 |

Table S2: Natural Charge, Natural Population of TS-keto-enol in gas phase at M062X/6 311++G(2d,2p)//B3LYP/6-31G(d,p).

| Natural Population | | | | Charge | Atom |
| --- | --- | --- | --- | --- | --- |
| Total | Rydberg | Valence | Core |  |  |
| 6.17155 | 0.01923 | 4.15311 | 1.99921 | -0.17155 | C1 |
| 6.22398 | 0.01934 | 4.20552 | 1.99912 | -0.22398 | C2 |
| 5.78804 | 0.02212 | 3.76687 | 1.99905 | 0.21196 | C3 |
| 6.13146 | 0.01932 | 4.11321 | 1.99893 | -0.13146 | C4 |
| 6.16699 | 0.02015 | 4.1477 | 1.99914 | -0.16699 | C5 |
| 6.22844 | 0.01989 | 4.20935 | 1.9992 | -0.22844 | C6 |
| 0.78791 | 0.00183 | 0.78608 | 0 | 0.21209 | H7 |
| 0.77929 | 0.00202 | 0.77726 | 0 | 0.22071 | H8 |
| 6.06694 | 0.03069 | 4.03756 | 1.99869 | -0.06694 | C9 |
| 0.76769 | 0.00219 | 0.7655 | 0 | 0.23231 | H10 |
| 0.78714 | 0.00199 | 0.78515 | 0 | 0.21286 | H11 |
| 6.14448 | 0.02421 | 4.12154 | 1.99872 | -0.14448 | C12 |
| 5.36121 | 0.03078 | 3.33131 | 1.99912 | 0.63879 | C13 |
| 5.78808 | 0.02679 | 3.76207 | 1.99921 | 0.21192 | C14 |
| 8.72488 | 0.02477 | 6.70031 | 1.99981 | -0.72488 | O15 |
| 15.76919 | 0.0301 | 5.73991 | 9.99919 | 0.23081 | S16 |
| 7.25119 | 0.03651 | 5.21541 | 1.99926 | -0.25119 | N17 |
| 6.69838 | 0.01596 | 4.68315 | 1.99927 | -0.69838 | C18 |
| 6.64918 | 0.01176 | 4.63815 | 1.99926 | -0.64918 | C19 |
| 7.62136 | 0.02675 | 5.59538 | 1.99923 | -0.62136 | N20 |
| 0.77054 | 0.00258 | 0.76796 | 0 | 0.22946 | H21 |
| 0.79178 | 0.00221 | 0.78958 | 0 | 0.20822 | H22 |
| 0.78002 | 0.00159 | 0.77843 | 0 | 0.21998 | H23 |
| 0.78198 | 0.00245 | 0.77952 | 0 | 0.21802 | H24 |
| 0.78253 | 0.00229 | 0.78024 | 0 | 0.21747 | H25 |
| 0.75806 | 0.0019 | 0.75616 | 0 | 0.24194 | H26 |
| 7.42898 | 0.02657 | 5.40306 | 1.99935 | -0.42898 | N27 |
| 0.49038 | 0.0046 | 0.48578 | 0 | 0.50962 | H28 |
| 6.24823 | 0.01791 | 4.2312 | 1.99912 | -0.24823 | C29 |
| 6.18604 | 0.01942 | 4.16741 | 1.99921 | -0.18604 | C30 |
| 6.24455 | 0.02038 | 4.22498 | 1.9992 | -0.24455 | C31 |
| 6.18189 | 0.01924 | 4.16344 | 1.99921 | -0.18189 | C32 |
| 6.25068 | 0.01825 | 4.23332 | 1.99911 | -0.25068 | C33 |
| 5.83021 | 0.0221 | 3.80906 | 1.99904 | 0.16979 | C34 |
| 0.79465 | 0.00201 | 0.79264 | 0 | 0.20535 | H35 |
| 0.79126 | 0.00189 | 0.78937 | 0 | 0.20874 | H36 |
| 0.79041 | 0.00187 | 0.78854 | 0 | 0.20959 | H37 |
| 0.79212 | 0.00191 | 0.79022 | 0 | 0.20788 | H38 |
| 0.77307 | 0.00216 | 0.77091 | 0 | 0.22693 | H39 |
| 0.62522 | 0.00412 | 0.6211 | 0 | 0.37478 | H40 |

Core 53.98066 (99.964% of 54)

Valence Lewis 111.35004 (95.991% of 116)

================== ============================

Total Lewis 165.33070 (97.253% of 170)

-----------------------------------------------------

Valence non-Lewis 4.32301 (2.543% of 170)

Rydberg non-Lewis 0.34630 (0.204% of 170)

================== ============================

Total non-Lewis 4.66930 (2.747% of 170)

Table S3: Natural Charge, Natural Population of TS-keto-enol in ethanol phase at M062X/6 311++G(2d,2p)//B3LYP/6-31G(d,p).

| Natural Population | | | | charge | Atom |
| --- | --- | --- | --- | --- | --- |
| Total | Rydberg | Valence | Core |  |  |
| 6.17192 | 0.01922 | 4.15348 | 1.99921 | -0.17192 | C1 |
| 6.22256 | 0.01938 | 4.20405 | 1.99912 | -0.22256 | C2 |
| 5.7884 | 0.02213 | 3.76721 | 1.99905 | 0.2116 | C3 |
| 6.13143 | 0.01925 | 4.11325 | 1.99893 | -0.13143 | C4 |
| 6.16742 | 0.02014 | 4.14815 | 1.99914 | -0.16742 | C5 |
| 6.22732 | 0.01989 | 4.20822 | 1.9992 | -0.22732 | C6 |
| 0.78777 | 0.00183 | 0.78595 | 0 | 0.21223 | H7 |
| 0.77872 | 0.00202 | 0.7767 | 0 | 0.22128 | H8 |
| 6.06743 | 0.03063 | 4.03811 | 1.99869 | -0.06743 | C9 |
| 0.76942 | 0.00218 | 0.76724 | 0 | 0.23058 | H10 |
| 0.78713 | 0.00198 | 0.78515 | 0 | 0.21287 | H11 |
| 6.14107 | 0.02458 | 4.11776 | 1.99872 | -0.14107 | C12 |
| 5.36575 | 0.03056 | 3.33609 | 1.9991 | 0.63425 | C13 |
| 5.78849 | 0.02669 | 3.76258 | 1.99921 | 0.21151 | C14 |
| 8.72666 | 0.02433 | 6.70251 | 1.99981 | -0.72666 | O15 |
| 15.76552 | 0.03017 | 5.73617 | 9.99919 | 0.23448 | S16 |
| 7.24709 | 0.03599 | 5.21184 | 1.99926 | -0.24709 | N17 |
| 6.69954 | 0.01587 | 4.6844 | 1.99927 | -0.69954 | C18 |
| 6.64829 | 0.01193 | 4.6371 | 1.99926 | -0.64829 | C19 |
| 7.61801 | 0.02711 | 5.59167 | 1.99922 | -0.61801 | N20 |
| 0.77545 | 0.00259 | 0.77285 | 0 | 0.22455 | H21 |
| 0.79108 | 0.00227 | 0.78881 | 0 | 0.20892 | H22 |
| 0.7789 | 0.00159 | 0.77731 | 0 | 0.2211 | H23 |
| 0.77805 | 0.00246 | 0.77559 | 0 | 0.22195 | H24 |
| 0.78347 | 0.00239 | 0.78107 | 0 | 0.21653 | H25 |
| 0.76291 | 0.00183 | 0.76108 | 0 | 0.23709 | H26 |
| 7.42863 | 0.02639 | 5.4029 | 1.99934 | -0.42863 | N27 |
| 0.48961 | 0.00462 | 0.48499 | 0 | 0.51039 | H28 |
| 6.24901 | 0.01786 | 4.23203 | 1.99912 | -0.24901 | C29 |
| 6.18595 | 0.01939 | 4.16734 | 1.99921 | -0.18595 | C30 |
| 6.24663 | 0.02042 | 4.22701 | 1.9992 | -0.24663 | C31 |
| 6.18119 | 0.01923 | 4.16274 | 1.99922 | -0.18119 | C32 |
| 6.25155 | 0.01819 | 4.23426 | 1.99911 | -0.25155 | C33 |
| 5.82864 | 0.02223 | 3.80736 | 1.99905 | 0.17136 | C34 |
| 0.7956 | 0.002 | 0.79359 | 0 | 0.2044 | H35 |
| 0.79145 | 0.00188 | 0.78957 | 0 | 0.20855 | H36 |
| 0.79046 | 0.00186 | 0.78859 | 0 | 0.20954 | H37 |
| 0.79256 | 0.00191 | 0.79065 | 0 | 0.20744 | H38 |
| 0.77385 | 0.00226 | 0.77159 | 0 | 0.22615 | H39 |
| 0.62501 | 0.00421 | 0.6208 | 0 | 0.37499 | H40 |

Core 53.98066 (99.964% of 54)

Valence Lewis 111.37178 (96.010% of 116)

================== ============================

Total Lewis 165.35244 (97.266% of 170)

-----------------------------------------------------

Valence non-Lewis 4.30158 (2.530% of 170)

Rydberg non-Lewis 0.34598 (0.204% of 170)

================== ============================

Total non-Lewis 4.64756 (2.734% of 170)

Table S4. First λ_max_ for 4-(methylsulfanyl)-3[(1Z)-1-(2 phenylhydrazinylidene) ethyl] quinoline-2(1H)-one using different functionals (model, acetonitrile)/6-311+G(d,p)//B3LYP/6-31G(d,p) level with different collection of solvation models (experimental value is 495 nm)

| **Model \functional** | **B3LYP** | **CAM-B3YP** | **PBE** | **PBE^a^** | **ωB97X-D** | **M06-2X** | **CIS** |
| --- | --- | --- | --- | --- | --- | --- | --- |
| **CPCM** | 449 (0.2825) | 425  (0.2177) | 415  (0.2115) | 416 (0.2115) | 424  (0.2006) | 424  (0.2158) | 363  (0.5873) |
| **SMD** | 447 (0.2875) | 422  (0.2176) | 500 (0.2241) | 502 (0.2235) ^b^ | 419  (0.2113) | 420  (0.2253) | 362  (0.6225) |
| **PCM** | 448 (0.2736) | 424  (0.2012) | 414  (0.2034) | 415 (0.2033) | 423  (0.1946) | 423  (0.2073) | 359 (0.5505) |
| **Gas phase** | 460 (0.2279) | 430  (0.1613) | 455  (0.1161) | 454  (0.1164) | 430  (0.1407) | 429  (0.1623) | 387  (0.2499) |

^a^PBE/6-311+G(2d,2p)

^b^ The result of PBE0/6-311+G(2d,2p) using SMD model is 436 nm (0.2739)
